# Using the taxon-specific genes for the taxonomic classification of bacterial genomes

**DOI:** 10.1186/s12864-015-1542-0

**Published:** 2015-05-20

**Authors:** Ankit Gupta, Vineet K Sharma

**Affiliations:** MetaInformatics Laboratory, Metagenomics and Systems Biology Group, Department of Biological Sciences, Indian Institute of Science Education and Research Bhopal, Madhya Pradesh, India

**Keywords:** Taxonomy, Phylogeny, Taxon-specific, Classification, Microtaxi, Taxonomic rank

## Abstract

**Background:**

The correct taxonomic assignment of bacterial genomes is a primary and challenging task. With the availability of whole genome sequences, the gene content based approaches appear promising in inferring the bacterial taxonomy. The complete genome sequencing of a bacterial genome often reveals a substantial number of unique genes present only in that genome which can be used for its taxonomic classification.

**Results:**

In this study, we have proposed a comprehensive method which uses the taxon-specific genes for the correct taxonomic assignment of existing and new bacterial genomes. The taxon-specific genes identified at each taxonomic rank have been successfully used for the taxonomic classification of 2,342 genomes present in the NCBI genomes, 36 newly sequenced genomes, and 17 genomes for which the complete taxonomy is not yet known. This approach has been implemented for the development of a tool ‘Microtaxi’ which can be used for the taxonomic assignment of complete bacterial genomes.

**Conclusion:**

The taxon-specific gene based approach provides an alternate valuable methodology to carry out the taxonomic classification of newly sequenced or existing bacterial genomes.

**Electronic supplementary material:**

The online version of this article (doi:10.1186/s12864-015-1542-0) contains supplementary material, which is available to authorized users.

## Background

The world-wide genome sequencing projects have been accelerated with the availability of high-throughput sequencing technologies. Several thousands of prokaryotic genomes have been sequenced or are currently being sequenced [1]. In this scenario, the taxonomic assignment and classification of a newly sequenced bacterial genome is one of the primary and significant tasks. Among the several available methods, DNA-DNA hybridization (DDH) and 16S rRNA gene based classification have been the key methods for the identification and taxonomic assignments of bacterial species [2]. The DNA-DNA hybridization is a molecular biology based technique which compares the genetic similarity between the DNA sequences of different species and the phylogenetic tree is constructed based on the observed similarity [3]. The phylogeny based classification is commonly carried out by the comparison of a highly conserved 16S rRNA gene which is a part of the small subunit of prokaryotic ribosome and is ubiquitously present in all prokaryotes [4].

Commonly, a species is defined as a set of strains with approximately 70% or greater DNA-DNA relatedness or 97% 16S rRNA identity [2]. Although these two methods are still the keystones of the present-day bacterial taxonomic classification, they have their own limitations. DDH is technically challenging, labor intensive and a time consuming method [5,6]. The DDH values lower than 50% cannot be used for estimating the genetic relatedness between distantly related species [7], and the information cannot be archived as a database [6]. In case of 16S rRNA gene sequence analysis, unlike DDH, the information is archival and can be used for various analyses [8]. Once the 16S rRNA gene sequence is determined it can be used as definitive comparative feature. Though the 16S rRNA gene sequence is capable of classifying a genome to the family or genus level, it is not very efficient in the differentiation of species [9]. For example, organisms with greater than 97% sequence identity may still belong to different species [9-11]. Multi-locus sequence analysis (MLSA) of housekeeping genes is another molecular method which has recently become popular for investigating taxonomic relationships [12-14]. MLSA of selected housekeeping genes accurately predicts the relationships between closely related genomes without the need for genome-wide comparison [15]. However, the main limitation in MLSA is the dependence on the choice of housekeeping genes which varies between different taxa [16].

Several computational approaches have been proposed for the taxonomic assignment of bacterial species. These approaches are primarily based on the comparison of gene order, gene content, average nucleotide identity (ANI) and nucleotide composition to determine the taxonomic relationships [16,17]. ANI can be considered as a computational substitute for DDH method. The homologous genomic regions shared between two genomes are represented by a mean of identity values [18,19]. Typically two genomes belonging to the same species show more than 95% identity using ANI which corresponds to more than 60-70% DDH values. The method has limited usability for the identity values less than 75%.

In another method, nucleotide composition is estimated using the dinucleotide and tetranucleotide frequencies and the trees based on the relative tetranucleotide frequencies corroborate well with the 16S rRNA based trees [20,21]. The presence and absence of protein-encoding gene families identified in sequenced genomes have also been used to determine the relationships between organisms [22,23]. The phylogenetic tree constructed using the gene order is suitable for resolving the phylogeny of closely related species, but offers poor resolution in case of distant species [24,25]. Though the above approaches are useful in estimating the genomic distance between the genomes or for constructing their phylogenies, they cannot be used for the systematic taxonomic classification of a genome in a taxonomic rank hierarchy from phylum to genus.

With the availability of whole genome sequences, the gene content based approaches appear more promising in inferring the bacterial taxonomy [26-28]. The complete set of genes present in all the strains of a particular species represents the ‘pan-genome’ of that species [29]. It includes the ‘core genome’ (present in all strains), and the ‘variable genome’ which includes the ‘dispensible genome’ (present in a few strains), and ‘unique genes’ (strain specific genes) [29]. In a study conducted by Welch et al., the genomes of three strains of *Escherichia coli* were compared. A total of 2,996 genes were found commonly present in all the three strains (core genome) and ~58% of the total genes were found only in one or two strains (variable genome) [30]. Another study using the gene content from 175 sequenced bacterial genomes showed that the classifications could be made only at the genus rank using this approach and not at higher taxonomic ranks [25].

In the current scenario where a large number of complete genome sequences of bacterial species are becoming available, the gene content based approaches could provide valuable alternatives. Furthermore, the genome annotation of newly sequenced genome often reveals a substantial number of unique protein-coding genes present only in that genome [29,31]. The presence of such genes can also be exploited for the taxonomic identification and classification of genomes. The identification of conserved sequence indels (CSIs) and conserved sequence proteins have been used in the past for the evolutionary and taxonomic studies of selected prokaryotic groups [32-35]. But, these studies are restricted to only a few taxonomic levels, however, in principle this approach can be extended to all taxonomic levels.

A few phylogenetic approaches are available, such as AMPHORA2 [36] and PhyloPhlAn [37], which use the universally conserved genes to infer taxonomy by constructing the evolutionary relationships. AMPHORA2, a pipeline for phylogenomic reference of bacterial genomes, is based on the identification of 31 phylogenetic marker genes from the given set of protein sequences and constructing the phylogenetic tree. Similarly, PhyloPhlAn generates high-resolution microbial phylogenies by identifying 400 marker genes and building a phylogenetic tree from the subsequences of these proteins. Another tool, MetaPhlAn, uses the clade specific-marker genes to estimate the relative abundance of microbial cells by mapping metagenomic reads against them [38]. However, this tool is limited for the community profiling and classification of metagenomic reads.

Given the above background, it is apparent that no approach or method is available to determine the complete (from phylum to genus level) taxonomy of a bacterial genome using its complete set of protein-coding genes. Therefore, in this study, we have proposed a comprehensive approach which uses the total set of protein-coding genes of a genome and identifies unique genes specific to each taxonomic rank for assigning the bacterial taxonomy. The method uses the available taxonomic information as reference taxonomy for the known genomes and further uses this information to identify the taxon-specific genes unique to each taxonomic rank. Based on the above approach, a tool ‘Microtaxi’ is also developed which can be used for the taxonomic classification of the existing and newly sequenced bacterial genomes.

## Results

### Assignment of eggNOG ids (NOGs)

While using the gene content as a method to assign taxonomy, the identification of orthologous genes and their classification into known orthologous groups is desired [25,39]. Additionally, in order to compare the gene content of different species, a unique gene symbol or id is required for each functional gene since there is enormous diversity in gene functions and ambiguity in the gene annotations. Therefore, all the genes present in a genome were classified into different orthologous groups and were annotated with unique NOGs by carrying out BLAST against the eggNOG v4.0 database [40]. NOGs could be assigned to 95.6% of the total genes by BLAST against the eggNOG database.

### Extraction of unique NOGs

The whole genome-based approaches which have been adopted so far to deduce the relationship between species are based on the comparison of the total gene content to identify the set of genes which are common between them [26-28,41]. However, approach adopted in the present study follows a converse methodology. It is based on the selection of unique NOGs present at a specific taxonomic rank such that these NOGs are not present at the same taxonomic rank in any other phylum.

To identify the unique NOGs, the NOGs of all bacterial genomes present in a phylum were clubbed together and sorted using an in-house Perl script. This resulted in a complete list of NOGs present in that phylum. The list of NOGs of each phylum was compared with the NOGs of all other phyla. Using this methodology, the list of NOGs which are unique for a phylum was obtained. Similarly, the unique NOGs for each taxonomic rank, i.e., class, order, family and genus, were extracted. For example, 8,603 NOGs were unique to Firmicutes phylum and were not present in any other phylum. Similarly, 3,870 NOGs were unique to Bacilli class of the above phylum and were not present in any other class of any other phylum. Using the above methodology, the lists of NOGs unique to each taxonomic rank were prepared. These taxon-specific NOGs were further used for the taxonomic classification of a bacterial genome. The summary of the abundance and distribution of NOGs across different phyla is provided in Additional file 1.

### Assigning taxonomy to a new genome

**Figure 1:**
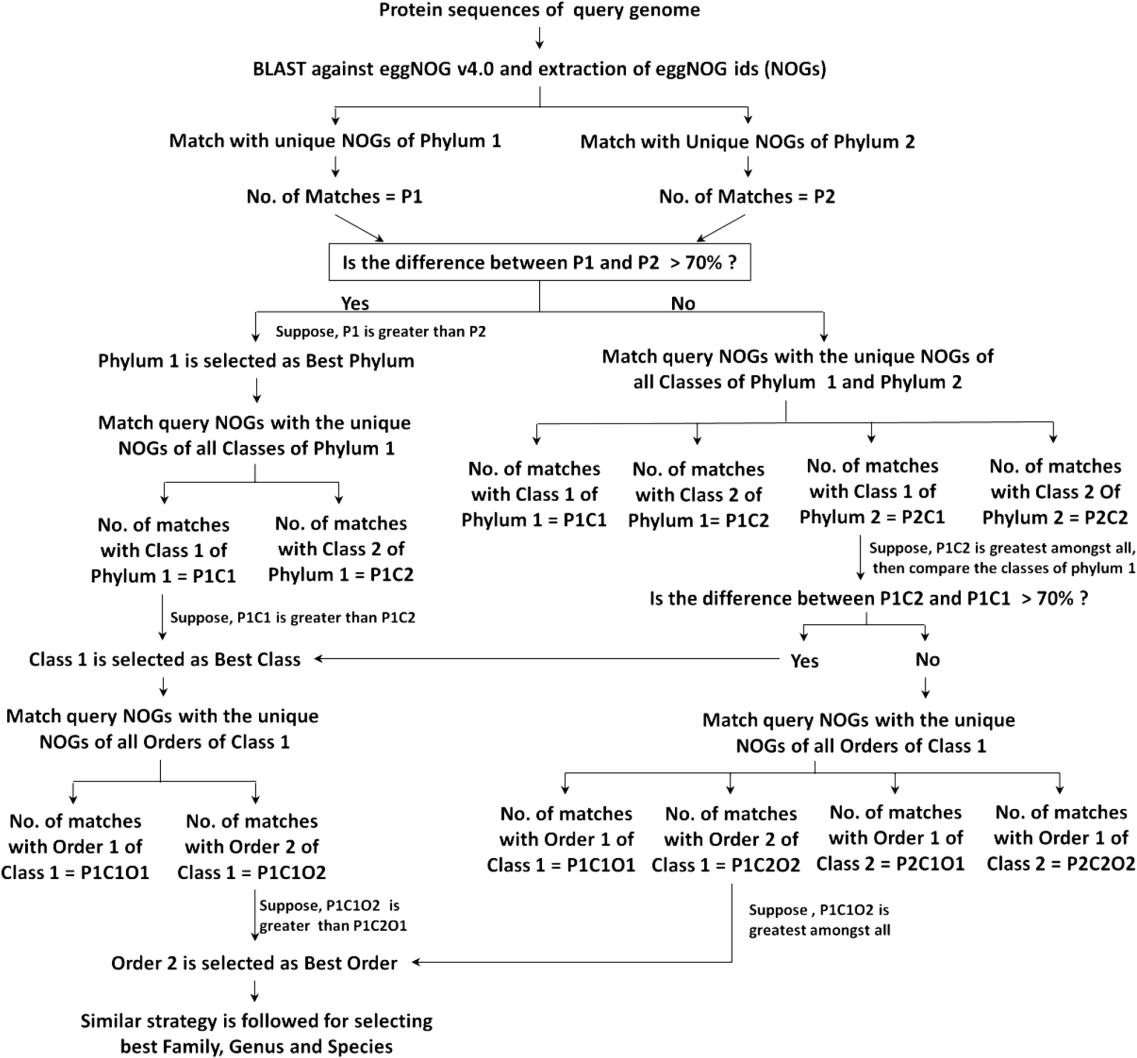
Flowchart of methodology used by Microtaxi.

To carry out the taxonomic assignment of a bacterial genome, the total set of proteins encoded by the genome is used. All protein sequences of the genome are assigned with NOGs by performing BLAST against the eggNOG 4.0 database (Figure 1). The list of unique NOGs of a query genome is compared against the list of unique NOGs of each phylum. The phylum which shows the maximum number of NOGs matches with the query genome is selected. For the selected phylum, the NOGs of each class present in that phylum are compared with the NOGs of the query genome and the class which shows the maximum number of matches is selected. Similar methodology is carried out to select the order, family, genus and species for the query genome.

In case, the second best phylum shows ≥30% NOGs matching with the NOGs of the best matched phylum, the best phylum is selected by comparing the NOGs of the query genome with the classes present in both the phyla. The class with maximum number of NOGs matches is selected as the best class and its corresponding phylum is selected as the best phylum. In case, the top two classes of the selected phylum shows ≥30% NOGs matches, best class is selected by comparing the NOGs of query genome with the unique NOGs of orders present in both the classes and the order with maximum number of matches is selected as best order and its corresponding class and phylum are selected as the best class and best phylum. If the order is correctly assigned, the lower taxonomic levels were assigned as per the methodology defined for a single best match (Figure 1). The above methodology was used to develop a computational tool ‘Microtaxi’ which can be used to determine the taxonomy of a bacterial genome using its complete set of protein sequences as the input.

### Performance of Microtaxi

Since only a small fraction (0.13-26.41%) of the total NOGs from any bacterial genome were selected in the list of taxon-specific NOGs; all 2,406 genomes could be used as self-test set to evaluate the prediction accuracy of Microtaxi. It could predict the correct taxonomy till the species rank for 2,342 genomes and till the genus rank for 2,361 genomes (Additional file 2). For the remaining 45 genomes it could correctly predict at order rank for 43 genomes and at family rank for 41 genomes.

On the first test set consisting of 56 bacterial genomes, it showed 100% accuracy of classification at phylum, class, order and family level and an accuracy of 96.30% at the genus level (Additional file 3). On the second test set consisting of 36 recently published bacterial genomes, it displayed 100% accuracy of classification till the order rank. 35 of the 36 genomes were correctly classified till the genus rank and for the remaining one genome the correct classification could be made only till the order rank (Additional file 4).

On the third test set consisting of 17 bacterial genomes for which the complete taxonomy is not yet known, Microtaxi could predict the taxonomic classification for all the genomes (Additional file 5). The classification of Microtaxi was found correct for 16 out of 17 genomes on comparing it with the available taxonomic rank of these genomes. Since, for these genomes the complete taxonomy is not known and there is no reference to compare and validate the accuracy of the predicted classification, the results were confirmed using the 16S rRNA sequences of the four classes, alpha, beta, gamma and delta, of the proteobacteria phylum which was one of the phyla present among the 17 selected bacterial genomes. Among the four classes, the *gamma_proteobacterium_HdN1* genome belonging to the gamma proteobacterium class was assigned as *Hahella_chejuensis_KCTC_2396* by Microtaxi and it was also the only species identified in its family Hahellaceae. Therefore, the confirmatory 16S rRNA analysis could not be performed for this class.

**Figure 2:**
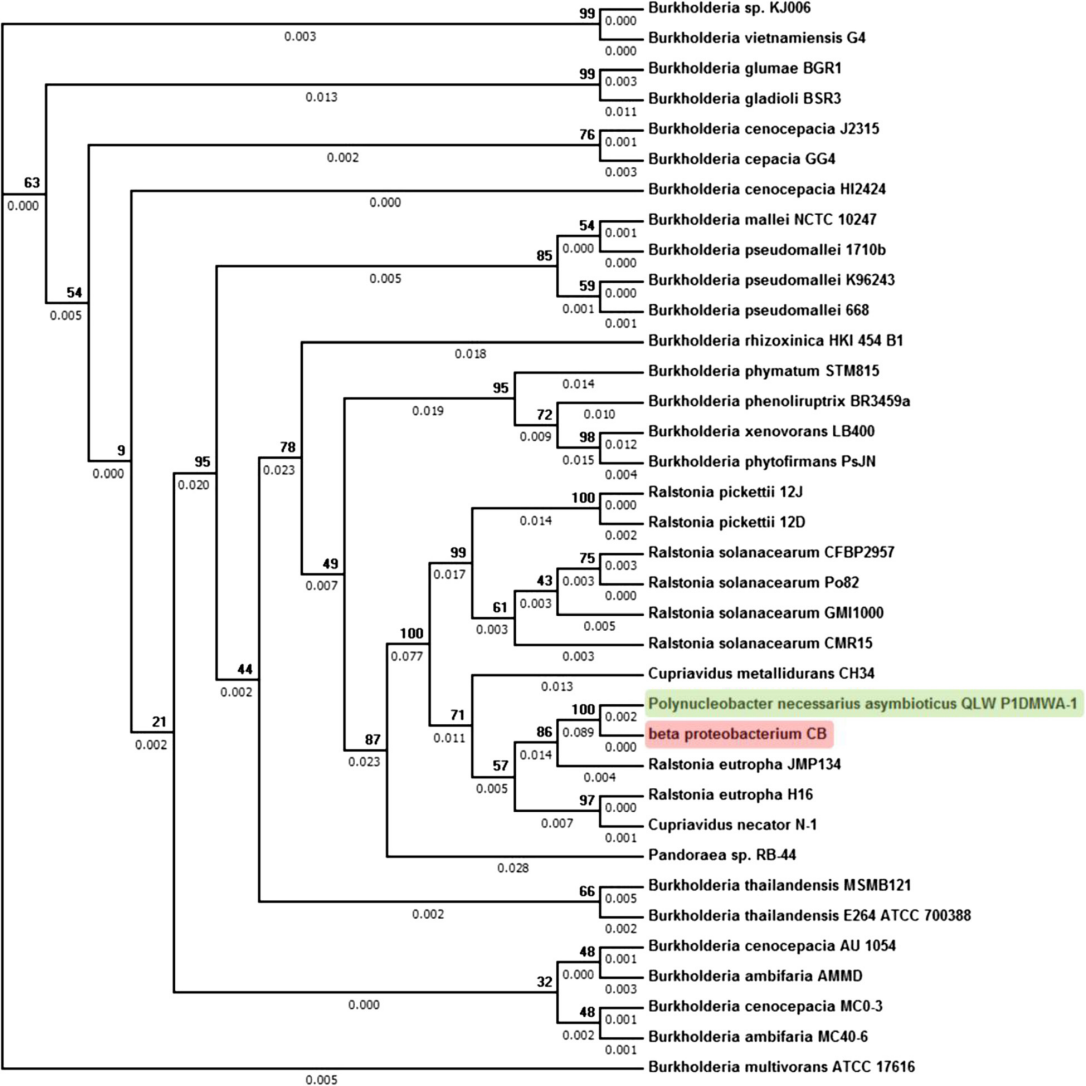
16S rRNA based phylogenetic tree of *Beta proteobacterium CB* and all the species of family Burkholderiaceae. Phylogenetic tree of *Beta proteobacterium CB *(highlighted in Red) indicates that it is nearest to *Plynucleobacter necessaries asymbioticus QLW P1DMWA-1 *(highlighted in Green). The percentage of replicate trees in which the associated taxa clustered together in the bootstrap test (1000 replicates) are shown next to the branches and the branch lengths are shown below the branches.

For the remaining three classes, 16S rRNA sequences were retrieved for all the strains of the predicted family included in the training dataset, since the prediction of microtaxi are shown to be 100% accurate up to the family level. For *Alpha proteobacterium HIM B59 and Delta proteobacterium BABL1*, the maximum identity achieved on alignment with other strains of their respective predicted families using 16S rRNA was only 82.1 and 77.2%. Hence, for these two genomes confirmatory 16S rRNA analysis was not performed since 16S rRNA analysis is not reliable at such low identity. However, in the case of *beta proteobacterium CB*, 16S rRNA showed >99% identity with the strains of the predicted family. Microtaxi classified this bacterium as *Polynucleobacter necessaries asymbioticus QLW P1DMWA-1.* Using the 16S rRNA sequences of 35 different species of the predicted family Burkholderiaceae and including the 16S rRNA of *beta proteobacterium CB*, the phylogenetic tree was constructed (Figure 2). The taxonomic tree shows *Beta proteobacteium CB* and *Polynucleobacter necessaries asymbioticus QLW P1DMWA-1* in the same clade which confirms the predictions made by Microtaxi.

### Functional analysis of unique NOGs

**Figure 3:**
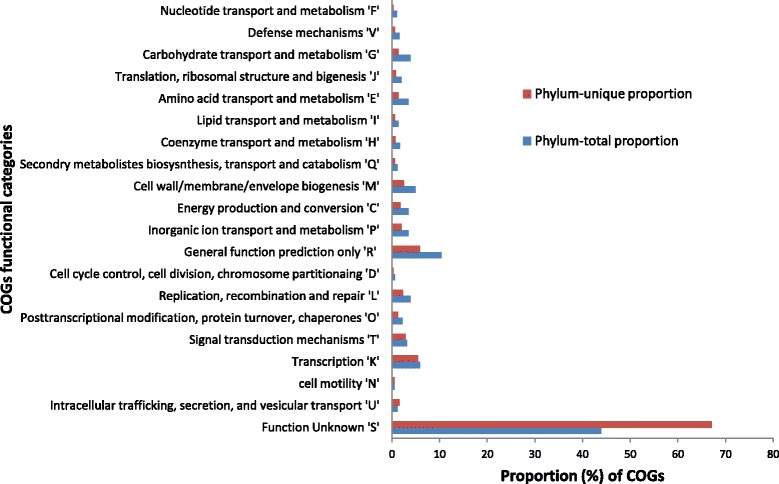
Proportion of COGs functional categories in phylum-total NOGs and phylum-unique NOGs.

The functional analysis was carried out by classifying all the NOGs identified in a phylum (phylum-total) in 23 COGs-based functional categories. Similarly, the phylum-unique NOGs were classified into the 23 functional categories to compare the proportion of functional categories in phylum-total and phylum-specific NOGs. It was observed that out of the 23 COGs functional categories, only ‘U’ and ‘S’ categories were significantly (*p ≤ 0*) overabundant (~1.4 and ~1.5 times, respectively) in the phylum-unique NOGs (Figure 3). The overabundance was calculated by dividing the observed proportion of phylum-unique NOG by the proportion of same NOG in the phylum-total set. The ‘S’ category was found to be overabundant in all phyla, whereas, the ‘U’ category showed more than 1.2 times abundance in only 17 out of the total 27 phyla. The other functional categories were under represented in the phylum-specific NOGs as compared to their phylum-total proportion.

The proteins belonging to the ‘U’ category are involved in intracellular trafficking, secretion, and vesicular transport functions which are essential for cellular processes and signaling. Studies have shown that such functions show species specificity, are often uniquely present in a phylum and also display a large sequence diversity across different bacterial phyla [42]. These functions are also shown to have a correlation with the organism’s lifestyle, environmental challenges and phylogenetic position [42]. The other category ‘S’ which represents proteins with unknown function was also found to be over-represented in the phylum-unique NOGS in all phyla (Additional file 6). The observed abundance of the proteins with ‘unknown function’ points toward an interesting aspect of the annotation methodology. The current methods of gene annotation are homology-based and thus those genes which show a significant similarity with a functionally annotated gene can be easily annotated. However, a gene which is unique to a species and does not have a close homolog in other species is likely to remain unannotated using homology-based annotation. Such genes would require functional characterization through experiments which is a time-consuming and tedious process. The abundance of functionally unknown genes in the phylum-specific set indicates that these genes are highly dissimilar in function to the known genes and have no close homolog. Thus, it appears that these genes might have evolved to meet the specific functional requirements of a species in a phylum and are unique to that phylum.

## Discussion

Apart from the commonly used methods such as DDH and 16S rRNA, the alternate methods are based on the comparison of the gene order or gene content of the genomes to carry out their taxonomic classification. The gene content of two species can be compared by identifying the common genes between the two species as the core genes and the genes unique to the two species as the peripheral genes or species specific genes. Thus, the gene content based methods use the proportion of core genes to identify the relationship between the species. However, the information of the species specific genes which are actually contributing to the uniqueness of that species is ignored in such approaches. Each species has some unique functions encoded by the unique genes of its genome and this information could be very useful for the identification and classification of the species and is successfully exploited in the current approach.

Furthermore, the core or common genes from two species provide information on the functions commonly present in the two species, and the number of core genes of any two species depends on the phylogenetic distance between the species. Two closely related genomes belonging to the same genus will have most of the genes in the core set. However, the total number of core genes will show a gradual reduction on moving to higher taxonomic ranks since the distance between the species increases. At the phylum level, which represents a distinct taxonomic lineage, the core set will contain only a handful of genes mostly comprising of essential and housekeeping genes. In addition, this core gene set for one phylum will also show considerable overlaps with the core gene set of other phyla since all genomes share a large fraction of genes, including essential genes, which are required for their survival in different environments. Therefore, the gene content based approach using core set will have limited application while carrying out the phylogenetic and taxonomic assignments.

In contrast, only a small number of genes will contribute to the species specific set of genes when two closely related species belonging to the same genus are compared. The number will show a gradual increase while moving from the genus to the phylum level. The complete set of genes derived from all the species belonging to a particular phylum represents the total repertoire of gene information present in that phylum. Now, if this total set of genes from one phylum is compared with the total set of genes in other phyla to remove the common genes, the set of phylum-specific genes can be obtained which are unique to that phylum and are not shared with other phyla. This set of genes has been used in this approach for the comprehensive and reliable classification of genomes.

Therefore, the underlying principle of the proposed approach is to carry out the taxonomic classification by exploiting the taxon-specific NOGs. The approach, implemented as Microtaxi tool, provides a new alternate methodology for predicting the taxonomy of a newly sequenced bacterial genome to the commonly used methodology using the 16S rRNA sequences. Using this approach, it is shown that the specific genes instead of the core genes can be used to determine the taxonomy of a bacterial genome. Since, this method is based on the available taxonomic classification information, its accuracy would also be limited by the accuracy of the available taxonomic information. In addition, like the 16S rRNA, the proposed approach using taxon-specific genes could provide classifications up to the genus rank and could also identify the closest known species to the query genome. After training on the known genome set, this method has been shown to perform exceptionally well on novel genomes (not included in the training data) which confirms the usability of this approach on the novel genomes. The performance of Microtaxi on different test datasets also attests to its prediction accuracy. The availability of new genomes would further improve the classification ability of Microtaxi.

## Conclusion

Since the approach provides a new alternate methodology to carry out the taxonomic classification of newly sequenced or existing bacterial genomes, the wide usage of this approach to determine the taxonomy of a novel bacterial genome is anticipated. The approach implemented as Microtaxi application is freely available as standalone program and web server at http://metagenomics.iiserb.ac.in/microtaxi and http://metabiosys.iiserb.ac.in/microtaxi.

## Methods

### Construction of database

Protein sequences of 2,420 bacterial genomes were retrieved from NCBI (ftp://ftp.ncbi.nlm.nih.gov/genomes/Bacteria/) and the complete taxonomic information for the genomes was retrieved from Greengenes database [43]. The taxonomic classification available at the Greengenes database was used as reference since the information is curated, free from chimeric sequences and provide standard taxonomic assignments [43]. Since the smallest number of genes known for a bacterial (*Mycoplasma genitalium*) genome is 475, only those (2,406) genomes which contained ≥475 genes were included in the final set [44]. To assign NOG to each gene, BLAST (v 2.2.26) was performed for the protein sequences of all 2,406 genomes against the eggNOG version 4.0 database which is a comprehensive (3,686 organisms) catalog of functionally annotated orthologous groups and deals with the existing problems of determining the orthology and provides an extensive and curated resource of orthologous groups of genes. Using the best hit of the BLAST results, the NOG for each protein sequence was extracted from the eggNOG database. Each NOG was included only once in a genome and thus the list of NOGs for each genome were prepared. The final curated dataset consisted of 27 bacterial phyla and 2,406 bacterial genomes belonging to 1,178 species (Additional file 7).

### Test datasets

Three test datasets were constructed to evaluate the performance of Microtaxi. In the first test set, 56 genomes were randomly selected from those genera for which at least nine bacterial genomes were known. The only reason for selecting the cut-off of nine genomes was that at this cut-off more than 50 genomes could be selected for the test dataset. Thus, 56 genomes were considered as test set and the remaining 2,350 genomes were used for extracting the taxon-specific NOGs. The second test set was constructed using the genomic information of 36 recently published complete bacterial genomes which were not present in the NCBI Genomes database used in this study. In the third test set, 17 bacterial genomes for which the complete taxonomy is not yet known were included.

### Other analysis

The phylogenetic trees using the 16S rRNA of selected classes belonging to Proteobacteria phylum were constructed by Maximum Likelihood method using RAxML software package [45]. Alignment of the 16S rRNA sequences was performed using CLUSTALW [46]. For functional classification and comparison of taxon-specific NOGs, all NOGs were classified into 23 functional COG categories by extracting their functional information from the eggNOGv4.0 database.

## Availability of supporting data

All the supporting data are included as additional files. The data of unique NOGs extracted for the different taxonomic lineages is available at the websites (metagenomics.iiserb.ac.in/microtaxi/download.php http://metabiosys.iiserb.ac.in/microtaxi) as NOGs_database.zip.

## Additional files

Additional file 1:
**Discussion and summary of the abundance and distribution of unique NOGs across different phyla.**
Additional file 2:
**Performance of Microtaxi on 2,406 bacterial genomes.**
Additional file 3:
**Performance of Microtaxi on the first test set consisting of 56 bacterial genomes.**
Additional file 4:
**Performance of Microtaxi on the second test set consisting of 36 recently published bacterial genomes.** p: phylum, c: class, o: order, f: family, g: genus, s: species, CND: Cannot Determine. **The prediction was incorrect in the case of *Eubacterium acidaminophilum DSM 3953* from family level. The correct classification is mentioned below: p__Firmicutes; c__Clostridia; o__Clostridiales; f__Ruminococcaceae; g__Eubacterium; For the above genome Microtaxi made the following prediction: p__Firmicutes; c__Clostridia; o__Clostridiales; f__Veillonellaceae; g__Acidaminococcus; Acidaminococcus_intestini_RyC_MR95_uid74445. Additional file 5:
**Performance of Microtaxi on 17 bacterial genomes for which the complete taxonomic classification is not available.** Note: The genomes which are highlighted in bold were selected for the analysis using 16S rRNA sequences and the phylogenetic tree for these cases is provided in Figure 2. Additional file 6:
**Proportion of COGs functional categories in phylum-total NOGs and phylum-unique NOGs for all the 27 phyla.**
Additional file 7:
**List of unique members at each taxonomic rank.**

## Abbreviations

DDH: DNA-DNA hybridization
MLSA: Multi-locus sequence alignment
ANI: Average nucleotide identity
CSI: Conserved sequence indels
NOG: eggNOG id
