# Supplementary material for: Using the taxon-specific genes for the taxonomic classification of bacterial genomes
Source: BMC Genomics. 2015 May 20;16(1):396. doi: 10.1186/s12864-015-1542-0 (PMC4438512; doi:10.1186/s12864-015-1542-0)
Supplement: Additional file 1: — Discussion and summary of the abundance and distribution of unique NOGs across different phyla. [file 12864_2015_1542_MOESM1_ESM.pdf]

Abundance and Distribution of unique NOGs

The accuracy of the approach of using the taxon-specific NOGs in determining the taxonomy of a bacterial genome is apparent from the results of Microtaxi on the above test datasets. Thus, it would be interesting to look at the abundance and functional significance of taxon-specific NOGs. Therefore, to examine if the unique NOGs identified in a particular phylum are present in all or most of the genomes present in that phylum, the NOGs which are present in more than 50% of the genomes belonging to a phylum were analyzed (Table A). It is observed that in six of the nine large phyla (>50 known genomes), namely, Proteobacteria, Firmicutes, Actinobacteria, Bacteroidetes, Tenericutes and Chloroflexi, only one to twelve unique NOGs are present in more than 50% of the total genomes present in that phylum. However, the remaining three large phyla, namely, Spirochaetes, Chlamydiae and Cyanobacteria have 48, 76 and 176 unique NOGs, respectively, present in more than 50% of the total genomes. It shows that as the number of known sequenced genomes increases in a phylum, the genomic diversity increases and, thus, only a few NOGs would show conservation across all genomes of the phylum. However, some phyla also showed a large number of unique NOGs present in more than half of their total genomes indicating that the functions represented by these unique NOGs might be important for achieving unique functional characteristics of the phyla. Distribution of these unique NOGs present in >50 % of the genomes was analyzed to examine if these are restricted to particular clades of species or are they distributed in all of the different classes and orders in a phylum. It was observed that these unique NOGs are universally distributed among the phylum and are not specific to a sub-clade as shown in the Table B below. The unique NOGs which were present in all genomes of a phylum and also had a known function are shown in Table C below. An example from the above Table is the 'AbrB family transcriptional regulator' (bactNOG81398) which is unique to the phylum cyanobacteria and is not present in any other phyla [1]. This protein is involved in nitrogen metabolism, toxin production, photosynthesis and oxidative stress which are the characteristic functions of the species belonging to the cyanobacteria phylum. Therefore, these unique NOGs are interesting candidates for further examination, since they are unique to the phylum and are present in all the genomes belonging to that phylum. However, it is an intensive task and requires considerable manual effort to search for the function of all the unique NOGs from the available literature. However, most of

these unique NOGs are still experimentally and computationally unannotated and hence it is not feasible to identify the functions of these NOGs. Therefore, the detailed functional examination of all unique NOGs could not be performed and presented in this study.

Table A: Distribution of NOGs in the 27 bacterial phyla

Phylum	Total Species	Total NOGs	# Unique NOGs	# NOGs with ³ 50% presence	# Unknowns in NOGs with ³ 50% presence (%)
Acidobacteria	6	9778	463	219	149 (68.04%)
Actinobacteria	271	53861	11139	12	7 (58.33%)
Aquificae	10	3835	290	118	69 (58.47%)
Armatimonadetes	1	2153	9	9	8 (88.89%)
Bacteroidetes	68	29750	4412	3	2 (66.67%)
Caldiserica	1	1269	2	2	0 (0.00%)
Chlamydiae	106	5251	313	76	46 (60.53%)
Chlorobi	12	7780	290	89	69 (77.53%)
Chloroflexi	17	11074	491	4	2 (50.00%)
Chrysiogenetes	1	2130	23	23	13 (56.52%)
Cyanobacteria	62	24276	2950	176	123 (69.89%)
Deferribacteres	2	2412	162	162	104 (64.20%)
Dictyoglomi	2	1882	5	5	3 (60.00%)
Fibrobacteres	2	2501	31	31	16 (51.61%)
Firmicutes	531	57959	8603	1	1 (100%)
Fusobacteria	6	5357	186	42	28 (66.67%)
Gemmatimonadetes	1	3044	24	24	14 (58.33%)
Nitrospirae	4	5394	55	8	6 (75.00%)
Planctomycetes	6	10445	1085	307	217 (70.68%)
Proteobacteria	1067	101269	32643	2	0 (0.00%)
Spirochaetes	58	17054	700	48	24 (50.00%)
Synergistetes	4	2931	234	145	96 (66.21%)
Tenericutes	131	24529	1210	1	0 (0.00%)
Thermi	18	9143	277	78	42 (53.85%)
Thermodesulfobacteria	1	1868	16	16	10 (62.50%)
Thermotogae	16	5220	405	107	64 (59.81%)
Verrucomicrobia	2	4520	345	245	238 (97.14%)

Table B: Distribution of unique NOGs with >50% abundance among different sub-groups of phyla.

PHYLUM	Class Coverage (%)	Order Coverage (%)	Family Coverage (%)	Genus Coverage (%)
Acidobacteria	100	100	100	100
Actinobacteria	100	60	94.12	85.25
Aquificae	100	100	100	100
Armatimonadetes	100	100	100	100
Bacteroidetes	100	100	83.33	89.74
Caldiserica	100	100	100	100
Chlamydiae	100	100	100	100
Chlorobi	100	100	100	100
Chloroflexi	100	100	100	100
Chrysiogenetes	100	100	100	100
Cyanobacteria	100	100	100	100
Deferribacteres	100	100	100	100
Dictyoglomi	100	100	100	100
Fibrobacteres	100	100	100	100
Firmicutes	50	28.57	25	18.84
Fusobacteria	100	100	100	100
Gemmatimonadetes	100	100	100	100
Nitrospirae	100	100	100	100
Planctomycetes	100	100	100	100
Proteobacteria	40	57.14	49.32	56.28
Spirochaetes	75	80	80	85.71
Synergistetes	100	100	100	100
Tenericutes	100	75	80	62.5
Thermi	100	100	100	100
Thermodesulfobacteria	100	100	100	100
Thermotogae	100	100	100	100
Verrucomicrobia	100	100	100	100

Table C: List of phylum-unique NOGs with known functions which are present in all the genomes of a specific phylum

Phylum	NOGs with known functions found conserved across all genomes
Acidobacteria	bactNOG261958, bactNOG256601, bactNOG242242, bactNOG213176, bactNOG171076, bactNOG166097
Armatimonadetes	bactNOG216685
Caldiserica	bactNOG11685
Chlamydiae	bactNOG95285, bactNOG94123, bactNOG252176, bactNOG249375, bactNOG167474, bactNOG162356, bactNOG121089, bactNOG101983
Chlorobi	bactNOG82661, bactNOG170115, bactNOG131518
Chrysiogenetes	bactNOG158055, bactNOG254807, bactNOG255668, bactNOG204973, bactNOG192028, bactNOG227284, bactNOG179593, bactNOG267012
Cyanobacteria	bactNOG93257, bactNOG86112, bactNOG82939, bactNOG81398, bactNOG159656, bactNOG141012, bactNOG140828, bactNOG140313, bactNOG140120, bactNOG119255, bactNOG11895, bactNOG08829
Deferribacteres	bactNOG92101, bactNOG90391, bactNOG38722, bactNOG260818, bactNOG252785, bactNOG252711, bactNOG252472, bactNOG249990, bactNOG248095, bactNOG247246, bactNOG247156, bactNOG246686, bactNOG245671, bactNOG243476, bactNOG241793, bactNOG240028, bactNOG238428, bactNOG238358, bactNOG236901, bactNOG231822, bactNOG192088, bactNOG184791, bactNOG179577, bactNOG170407, bactNOG149174, bactNOG13743, bactNOG131677, bactNOG131061, bactNOG127568, bactNOG117520
Dictyoglomi	bactNOG227153
Fibrobacteres	bactNOG268917, bactNOG268809, bactNOG267236, bactNOG266618, bactNOG262828, bactNOG256090, bactNOG224112, bactNOG220093, bactNOG166838, bactNOG155575
Gemmatimonadetes	bactNOG225178, bactNOG256905, bactNOG252300, bactNOG226252, bactNOG239699, bactNOG223488, bactNOG266385, bactNOG225592, bactNOG227470, bactNOG218739
Planctomycetes	bactNOG252691, bactNOG247080, bactNOG246595, bactNOG245717, bactNOG231713, bactNOG17162, bactNOG151592, bactNOG137839, bactNOG135679
Synergistetes	bactNOG252971, bactNOG251408, bactNOG248876, bactNOG247943, bactNOG241742, bactNOG241289, bactNOG164719, bactNOG14493, bactNOG137076, bactNOG107510, bactNOG105331
Thermi	bactNOG96955, bactNOG58199, bactNOG251092, bactNOG201877, bactNOG190973, bactNOG129316, bactNOG117290,
Thermodesulfobacteria	bactNOG265574, bactNOG256981, bactNOG206688, bactNOG256582, bactNOG210604
Thermotogae	bactNOG86596, bactNOG155123, bactNOG138453, bactNOG114539
Verrucomicrobia	bactNOG74590, bactNOG252414, bactNOG161646, bactNOG146672

Reference:

1. Larsson J, Nylander JA, Bergman B: **Genome fluctuations in cyanobacteria reflect evolutionary, developmental and adaptive traits**. *BMC evolutionary biology* 2011, **11**:187.
