# Supplementary figures and images for: Using the taxon-specific genes for the taxonomic classification of bacterial genomes
Source: BMC Genomics. 2015 May 20;16(1):396. doi: 10.1186/s12864-015-1542-0 (PMC4438512; doi:10.1186/s12864-015-1542-0)

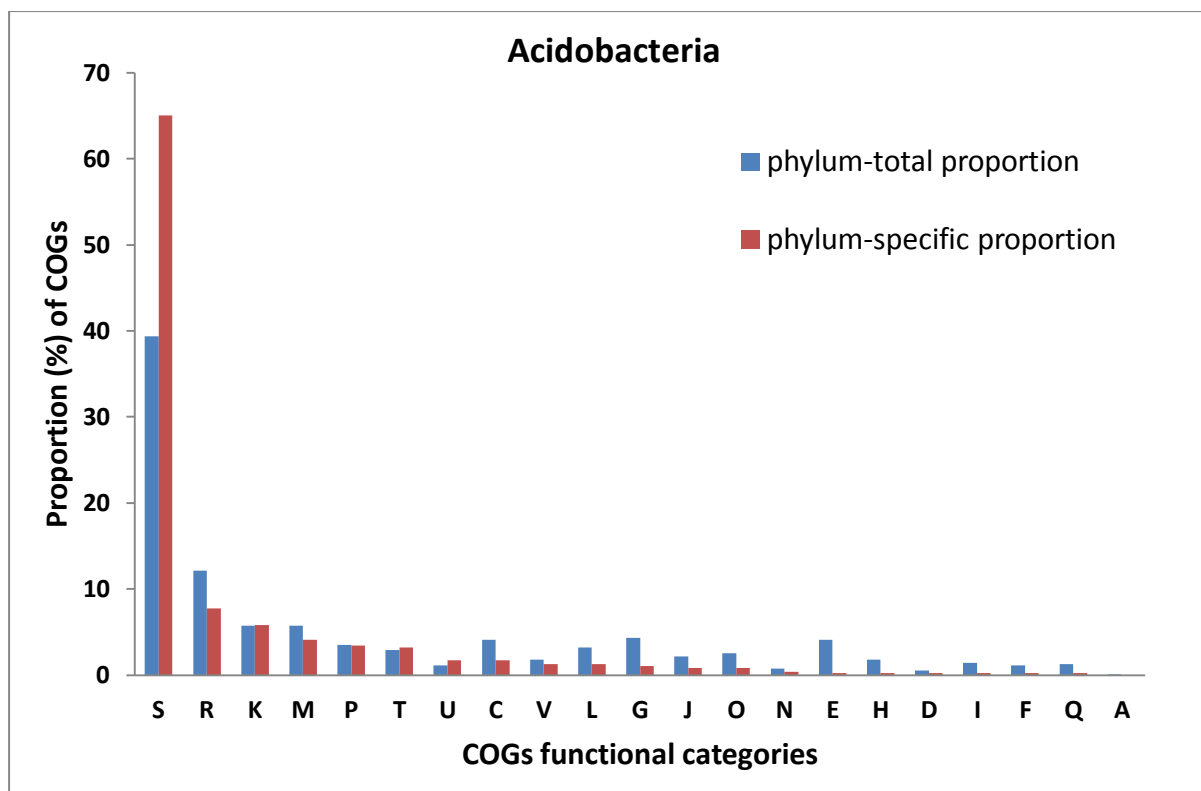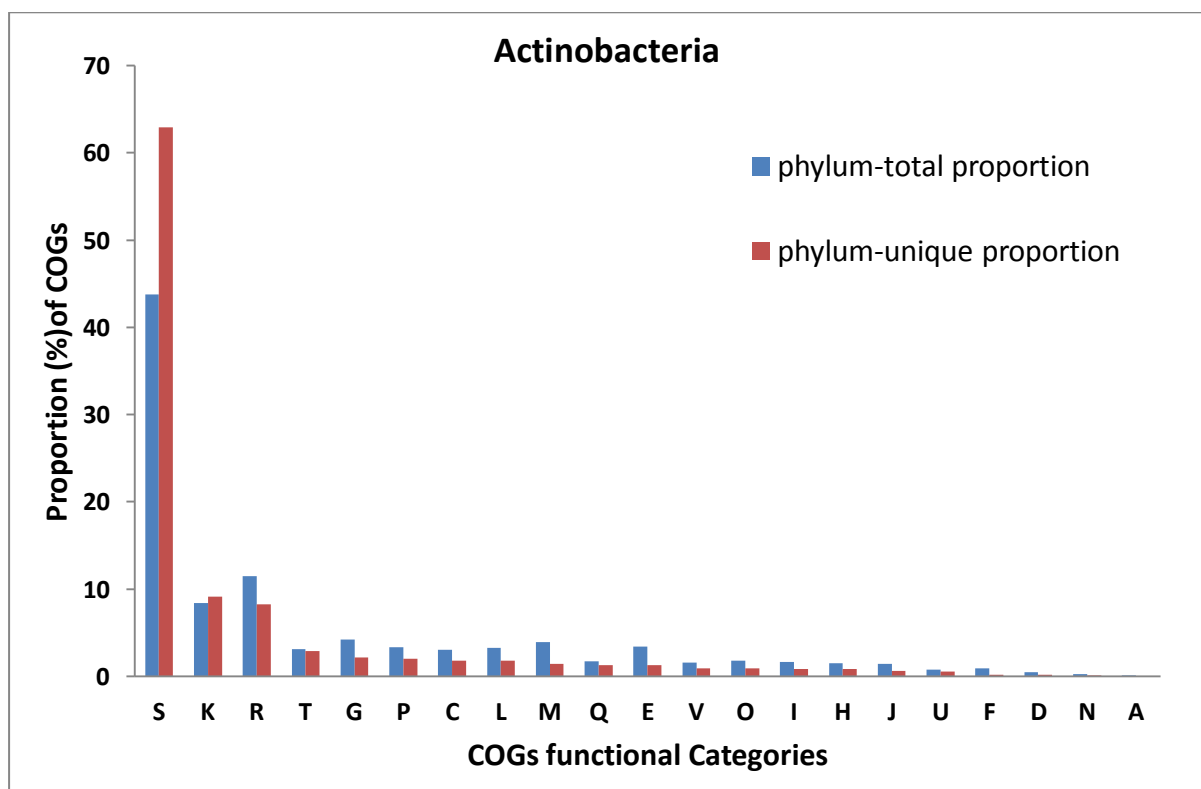

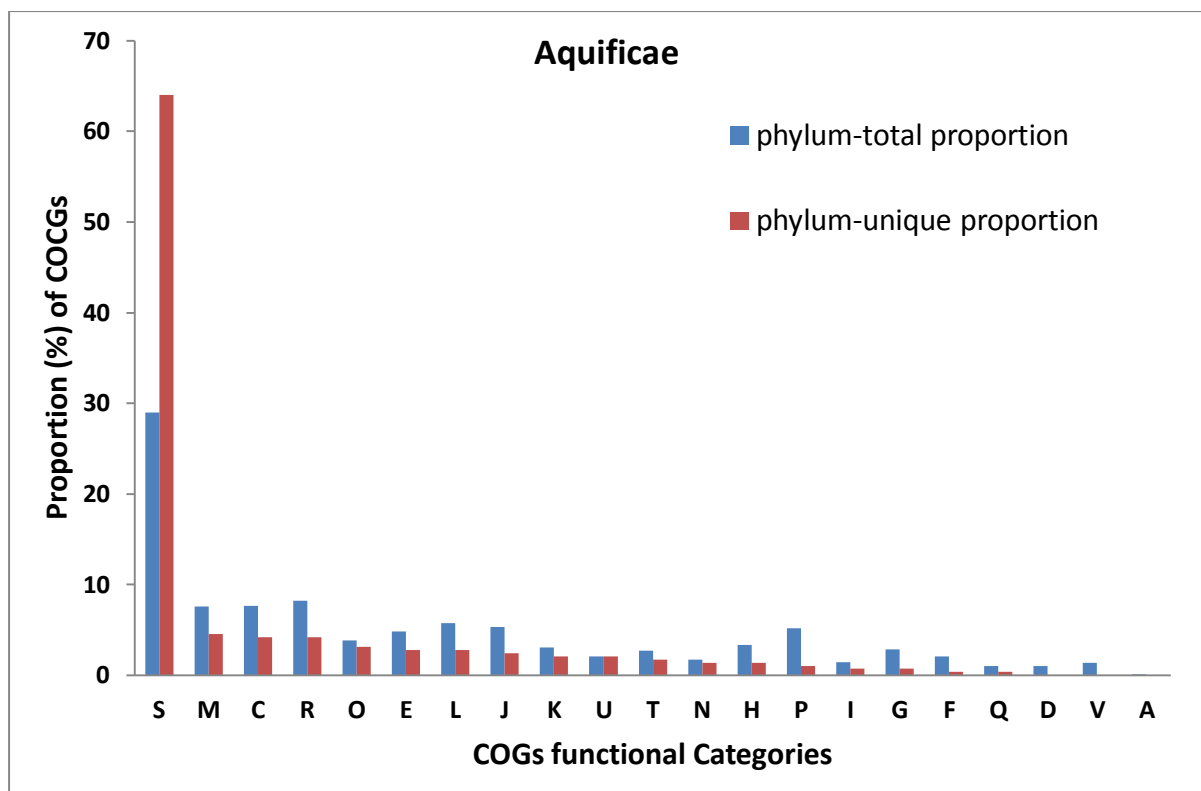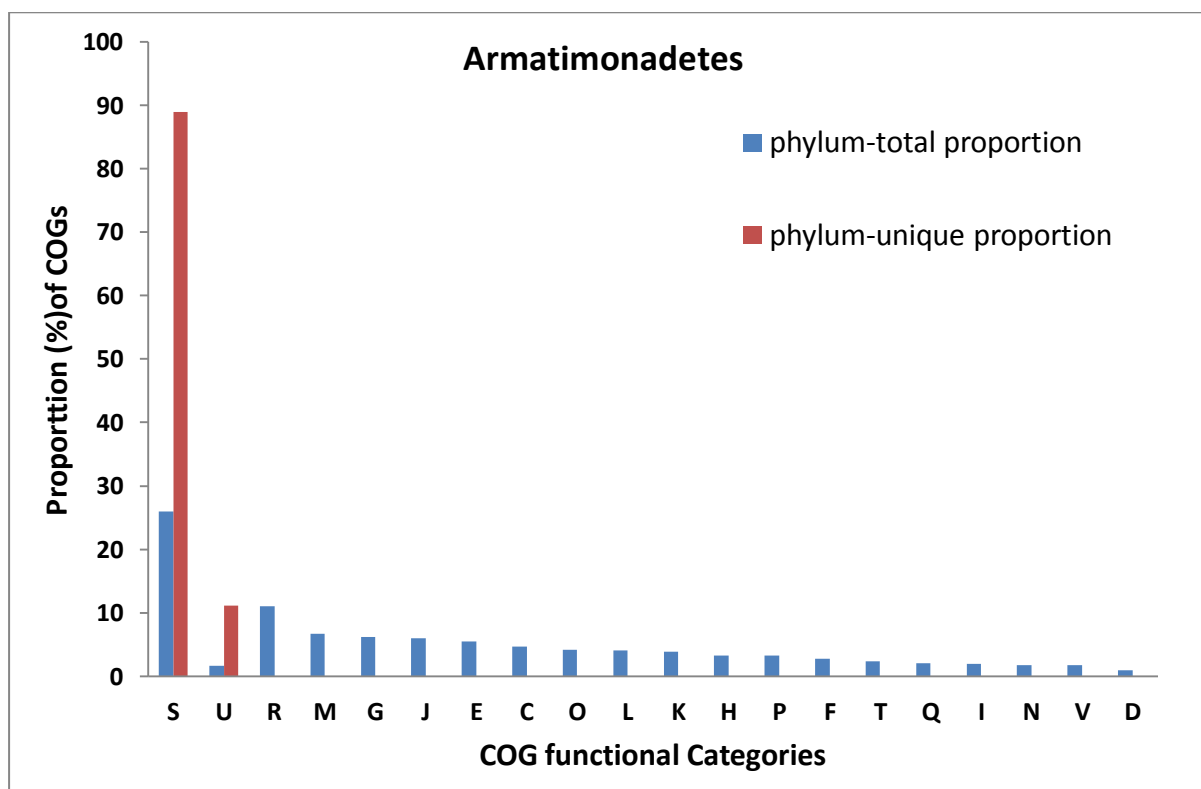

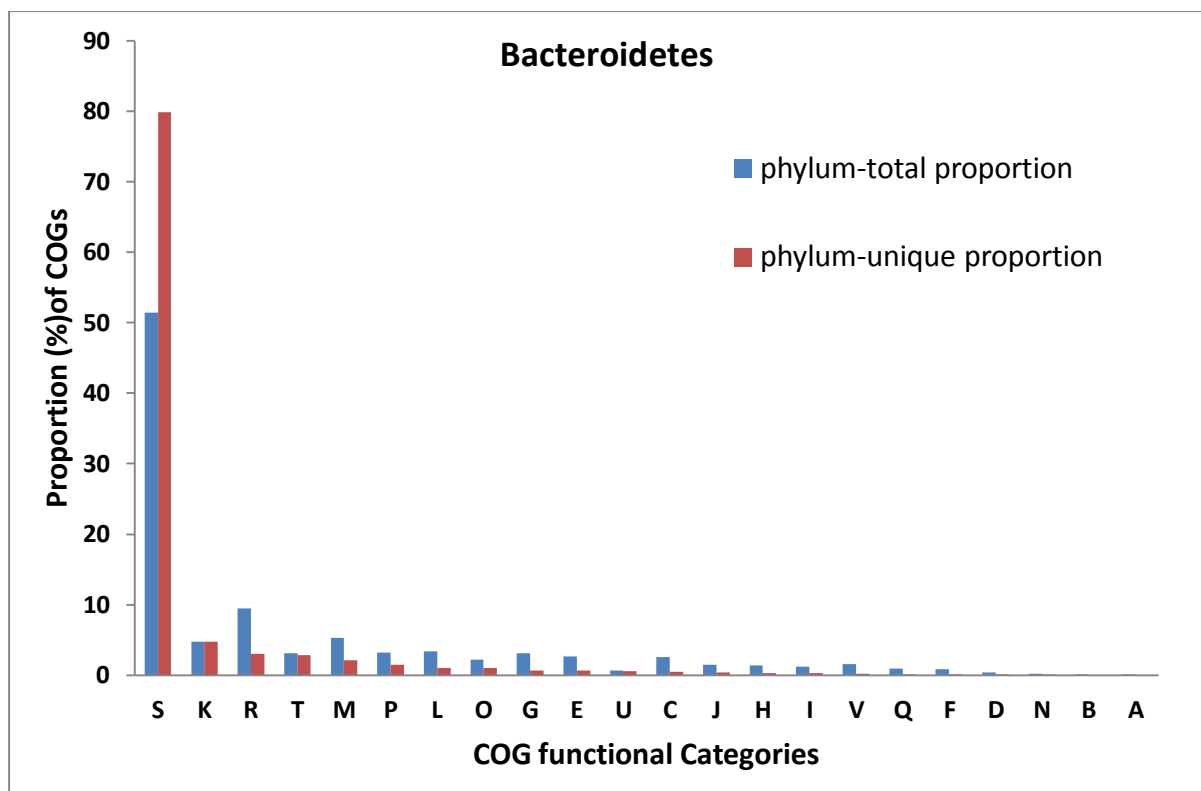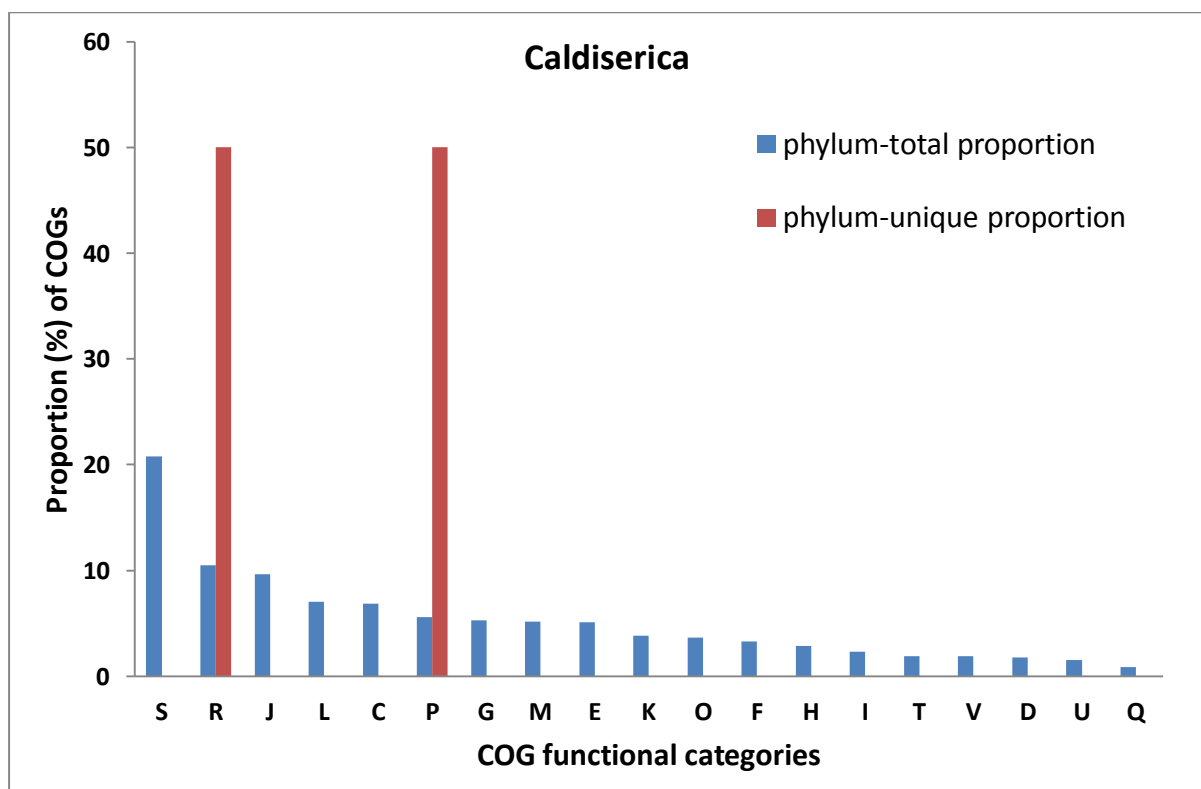

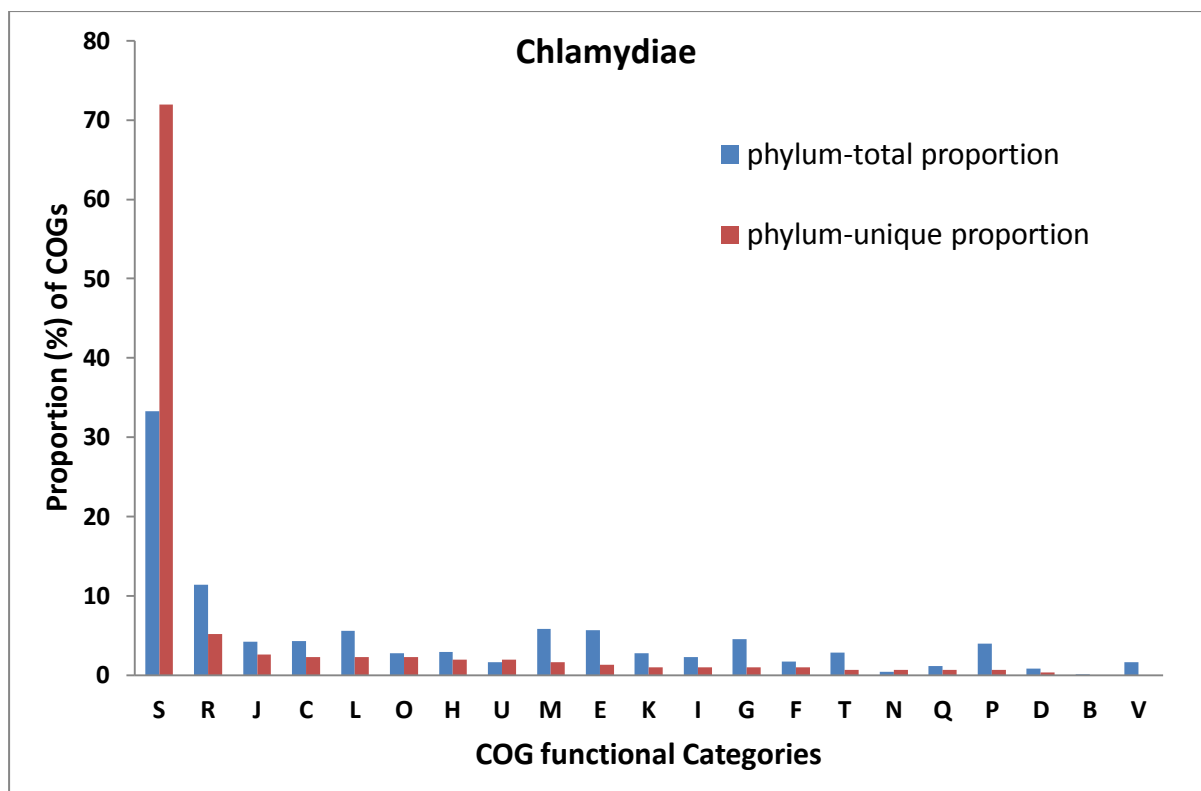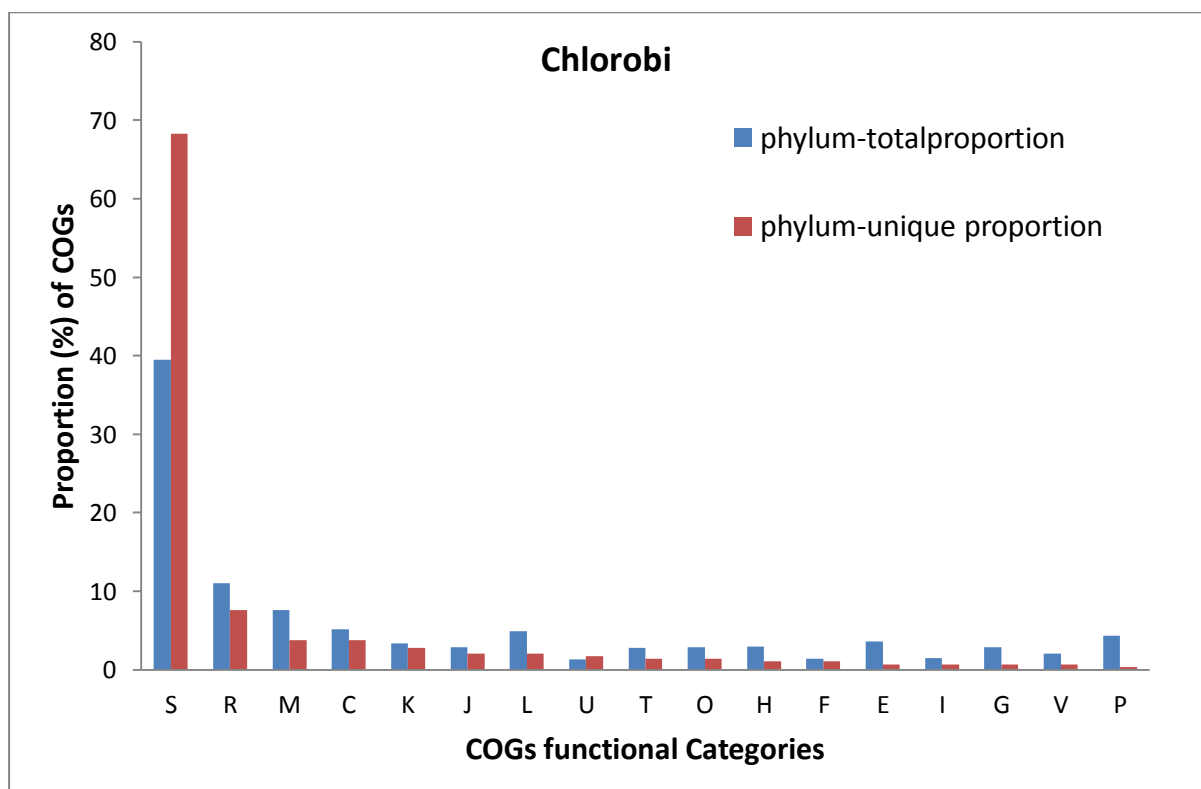

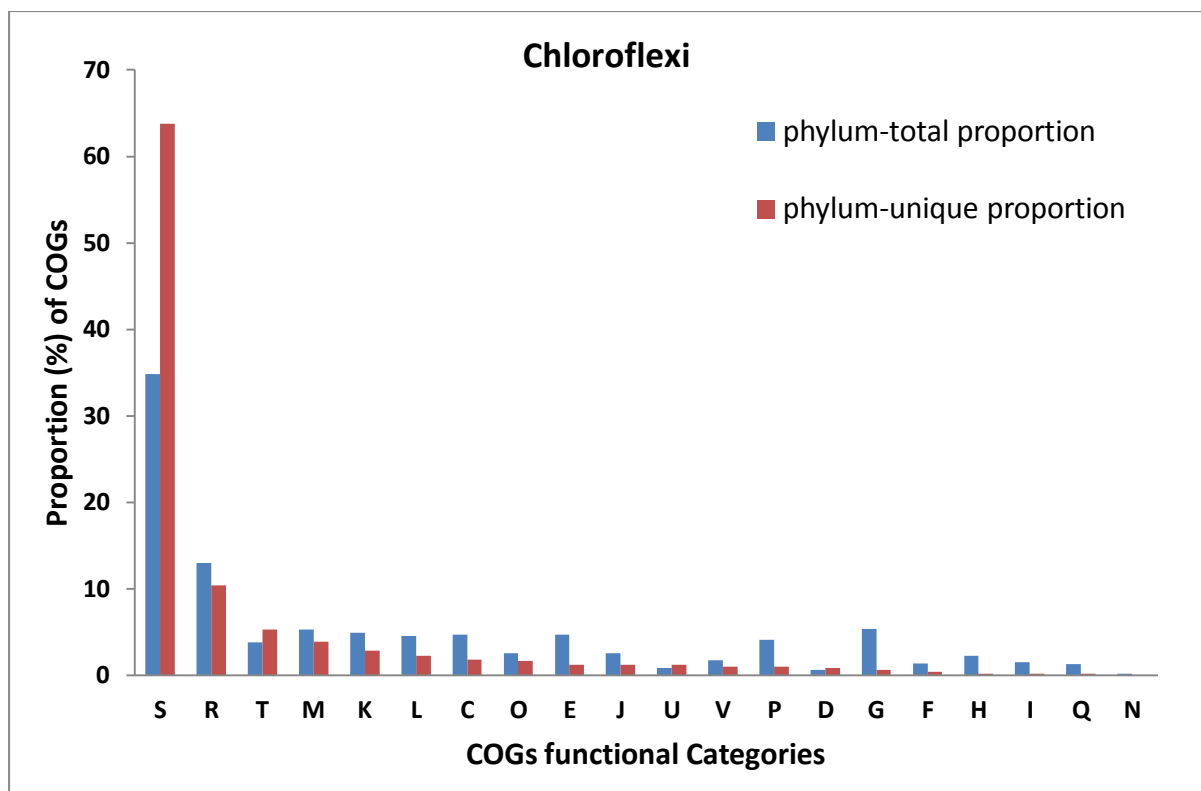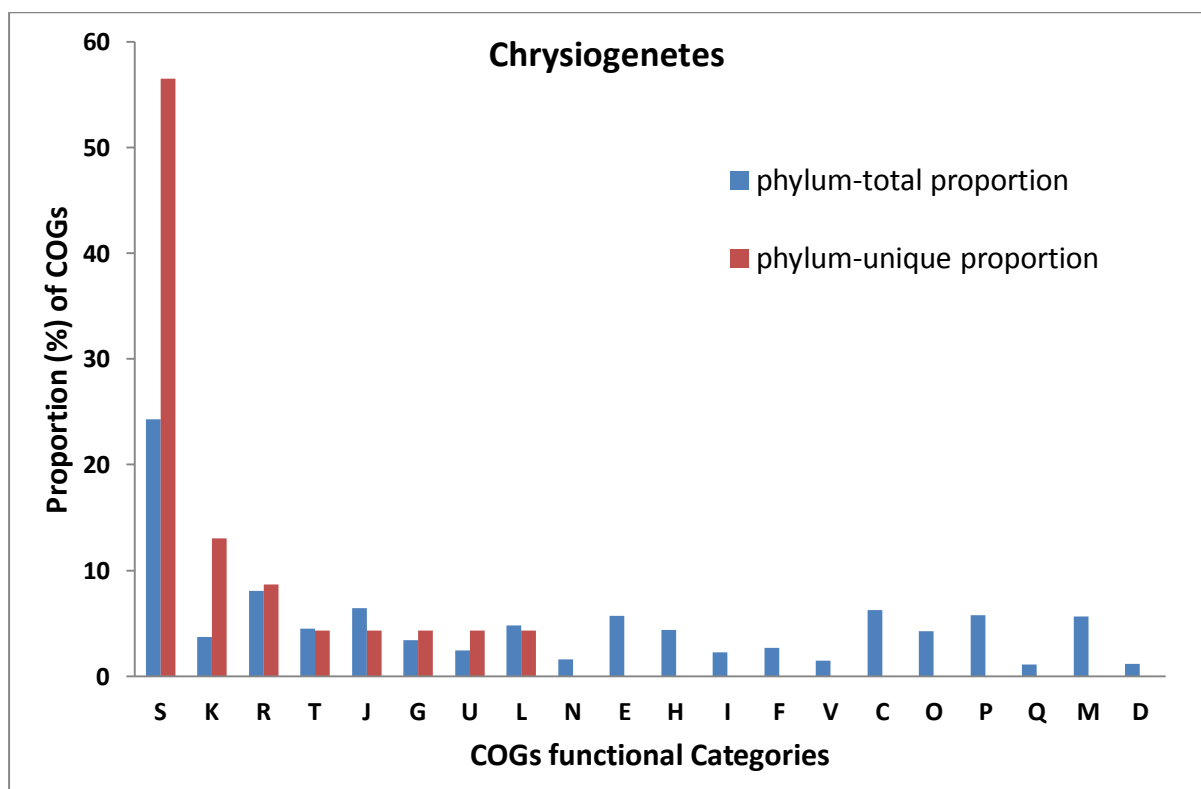

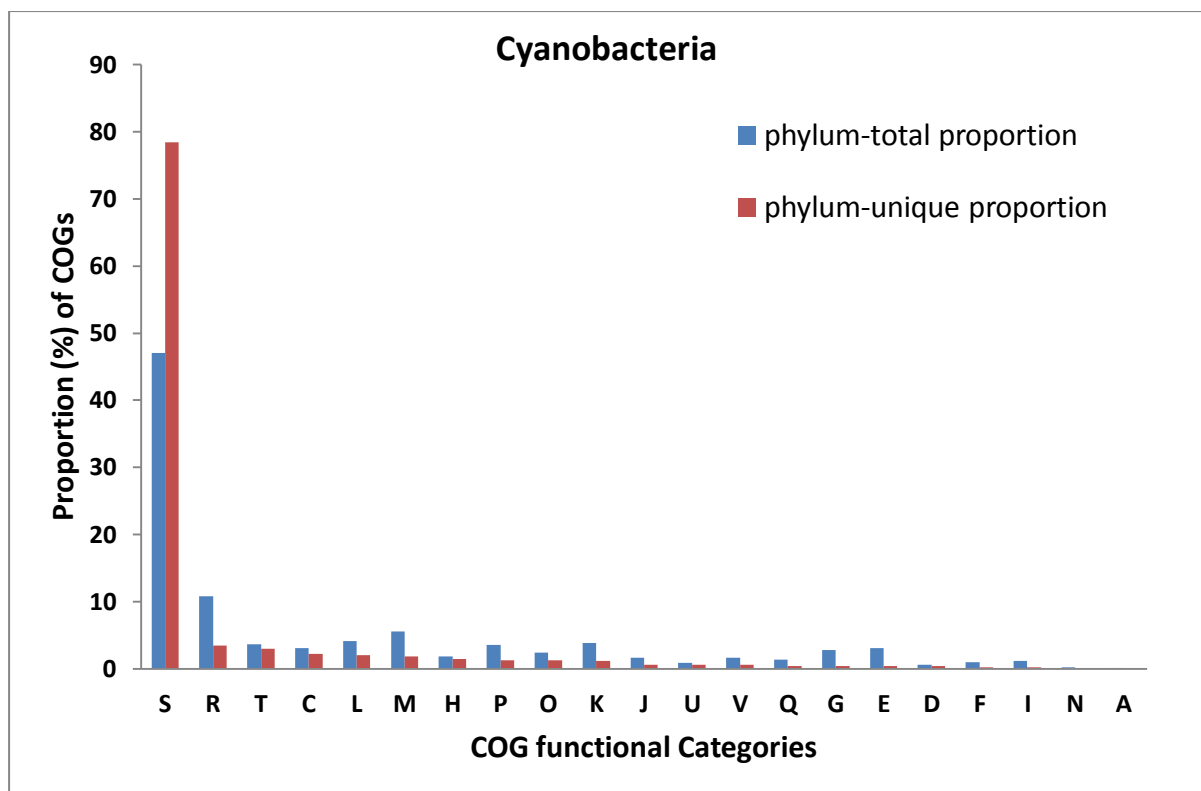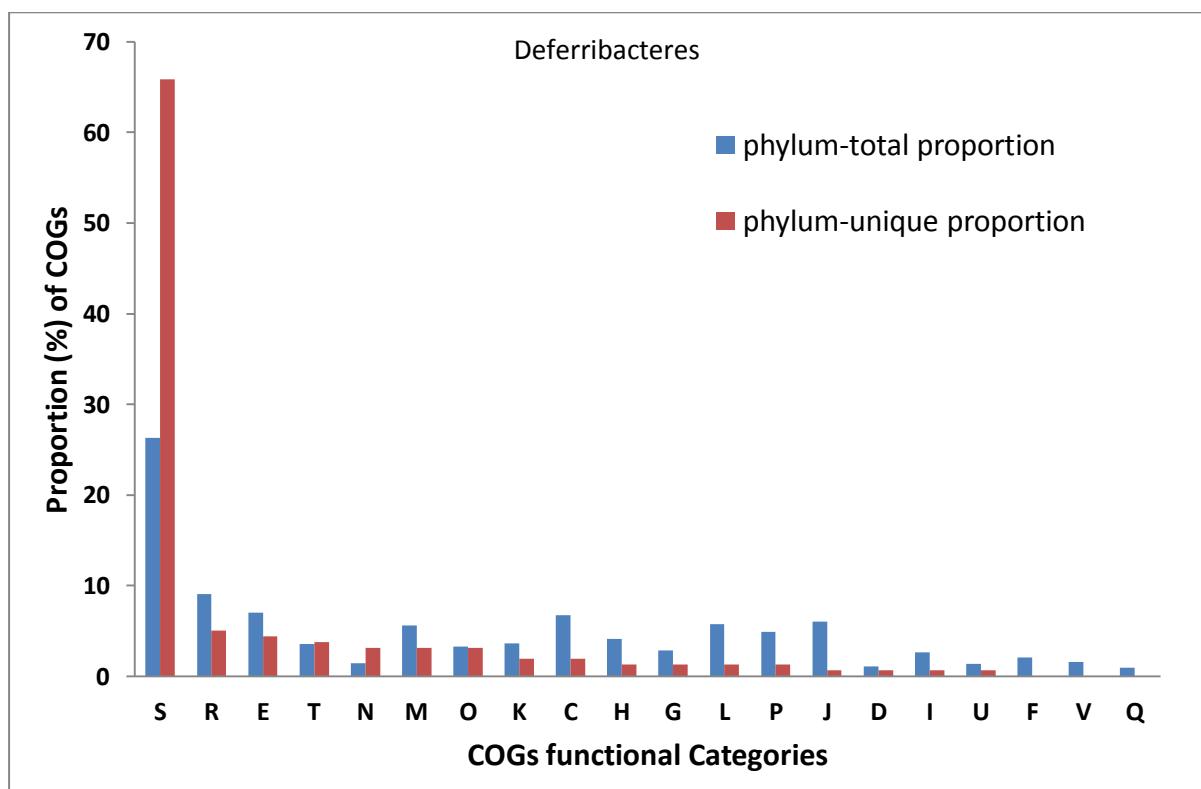

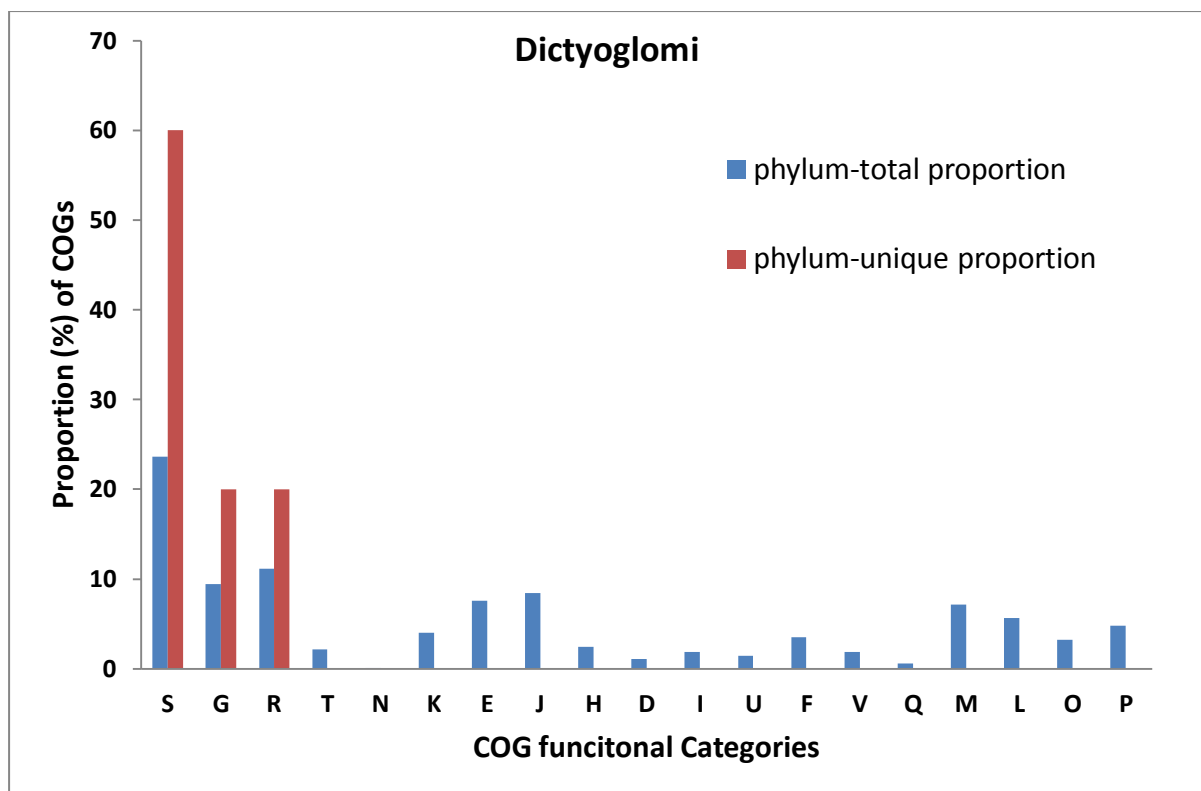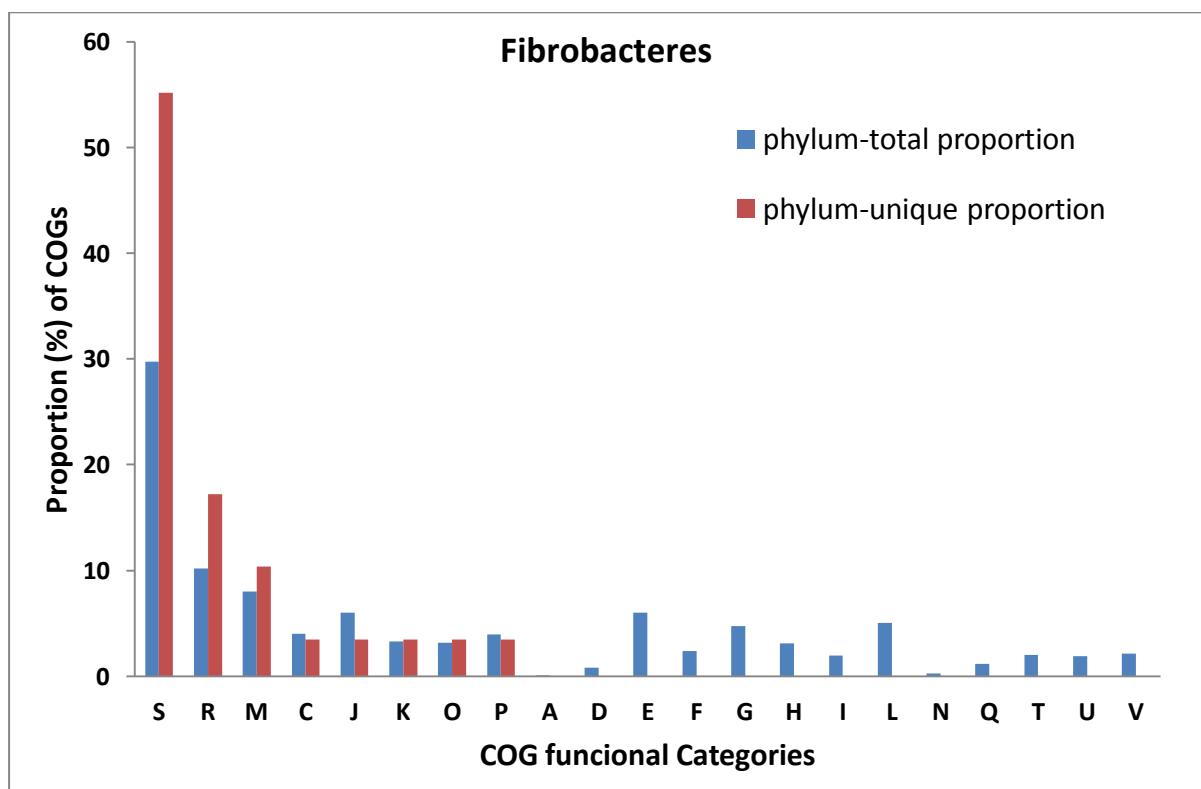

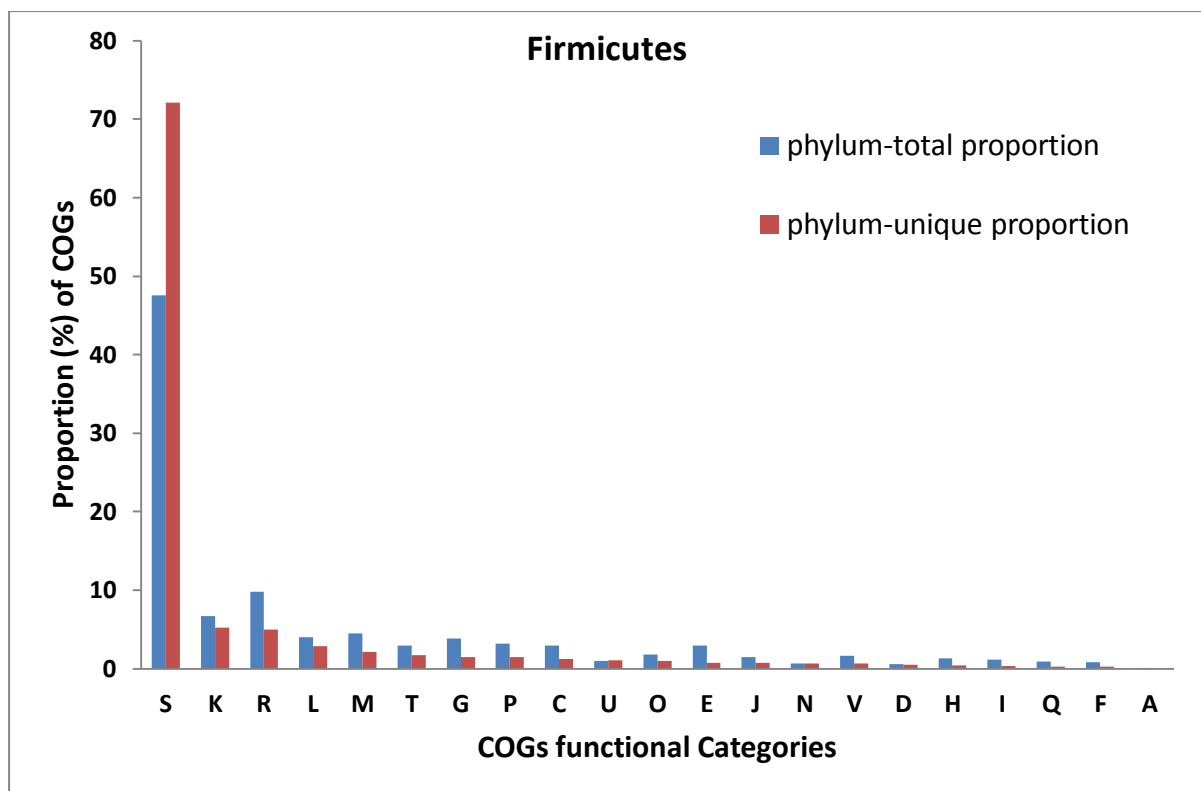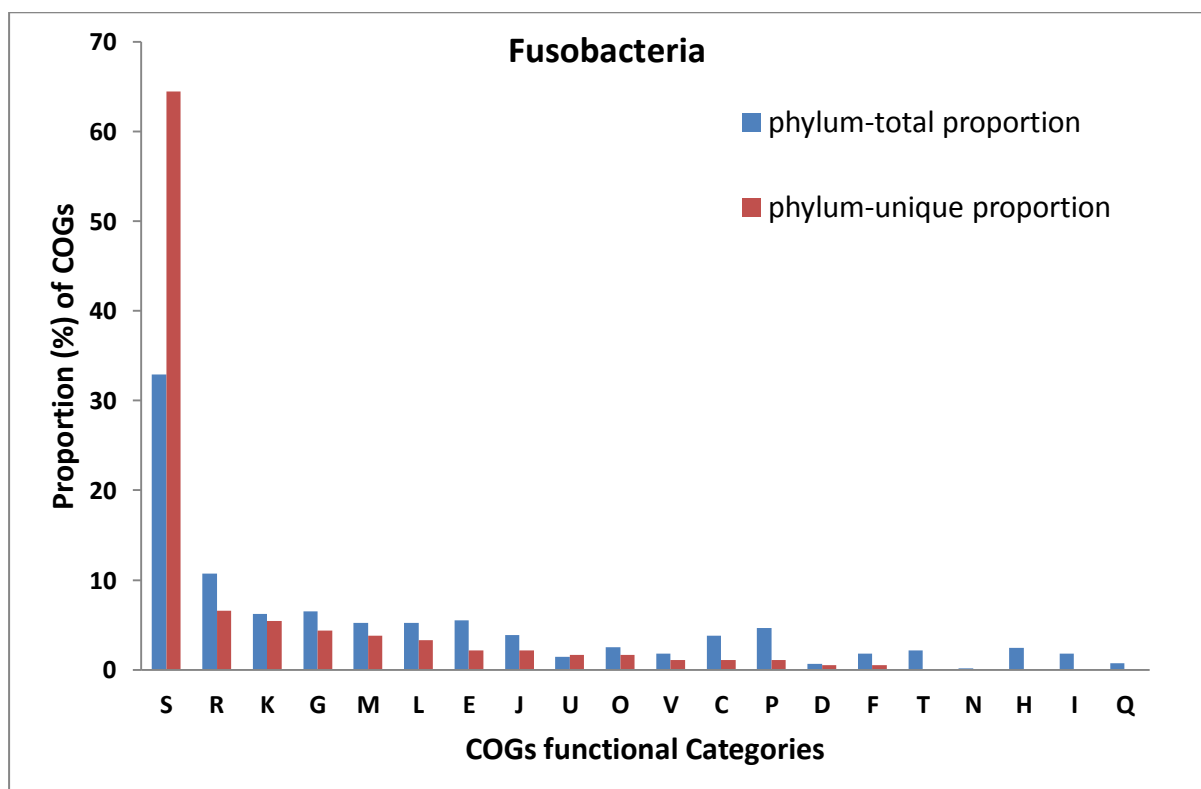

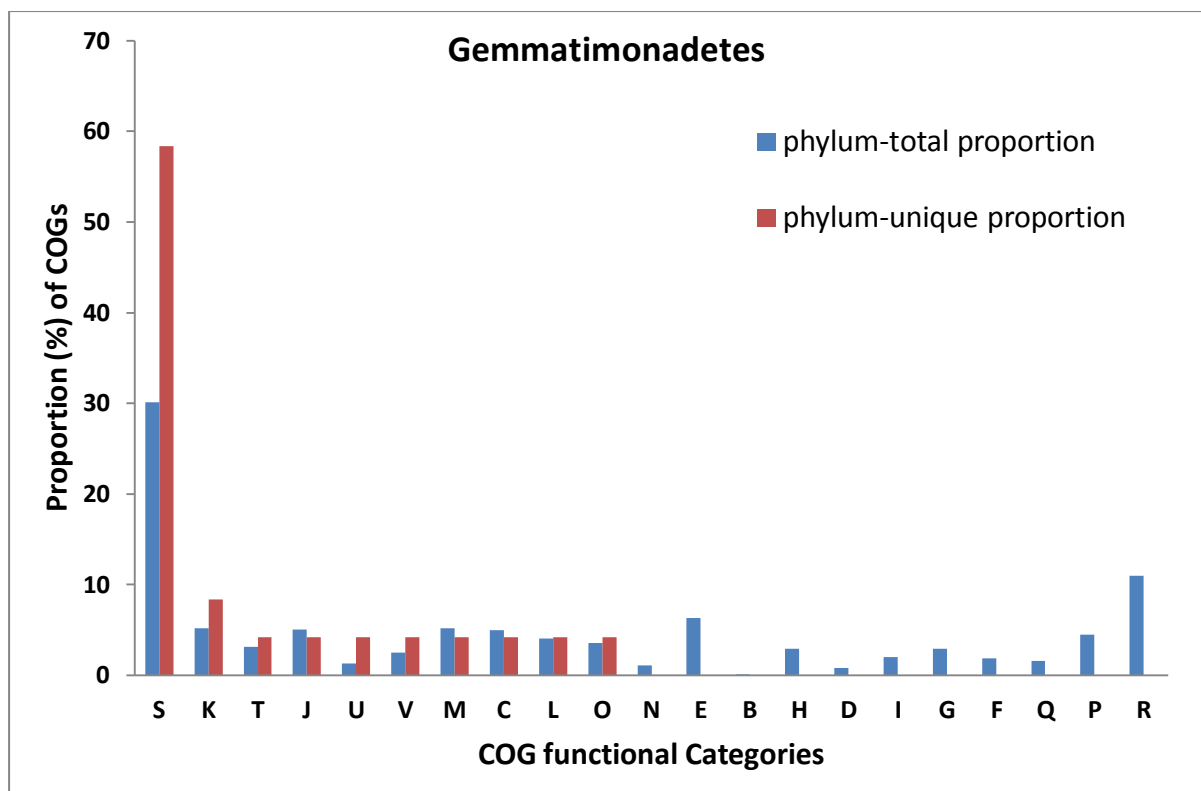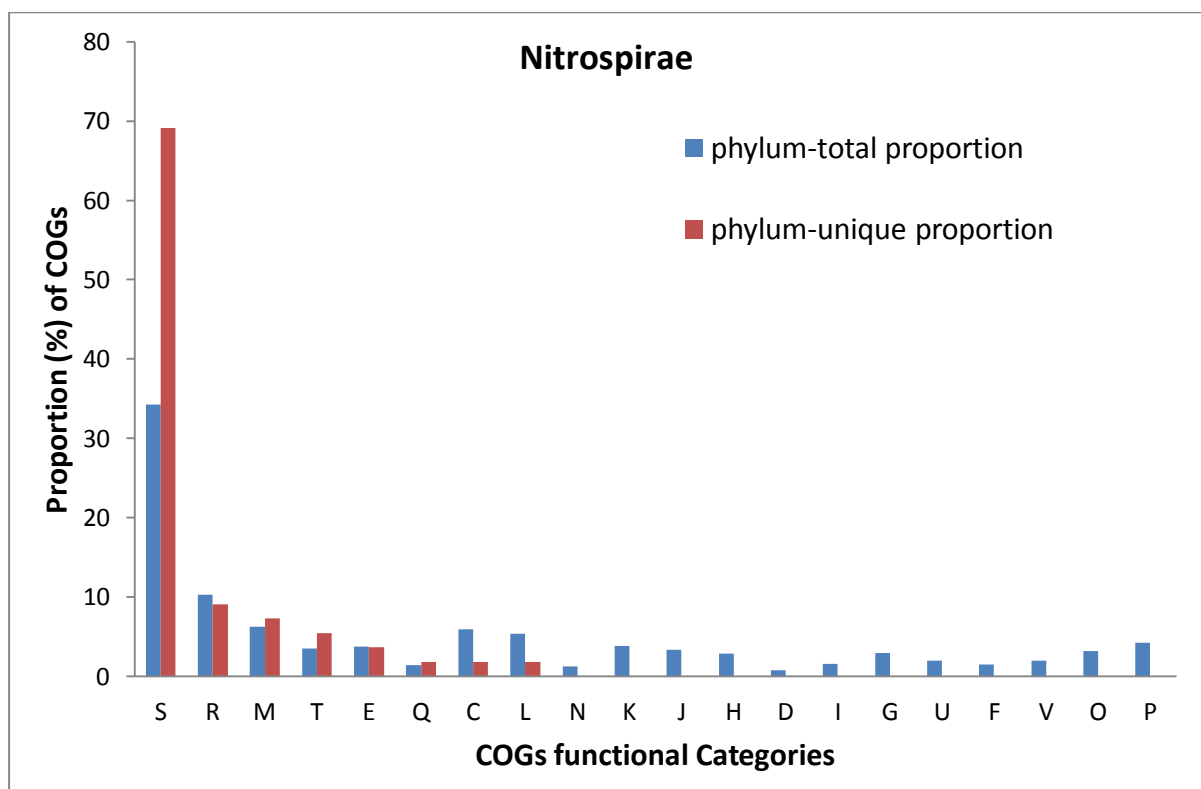

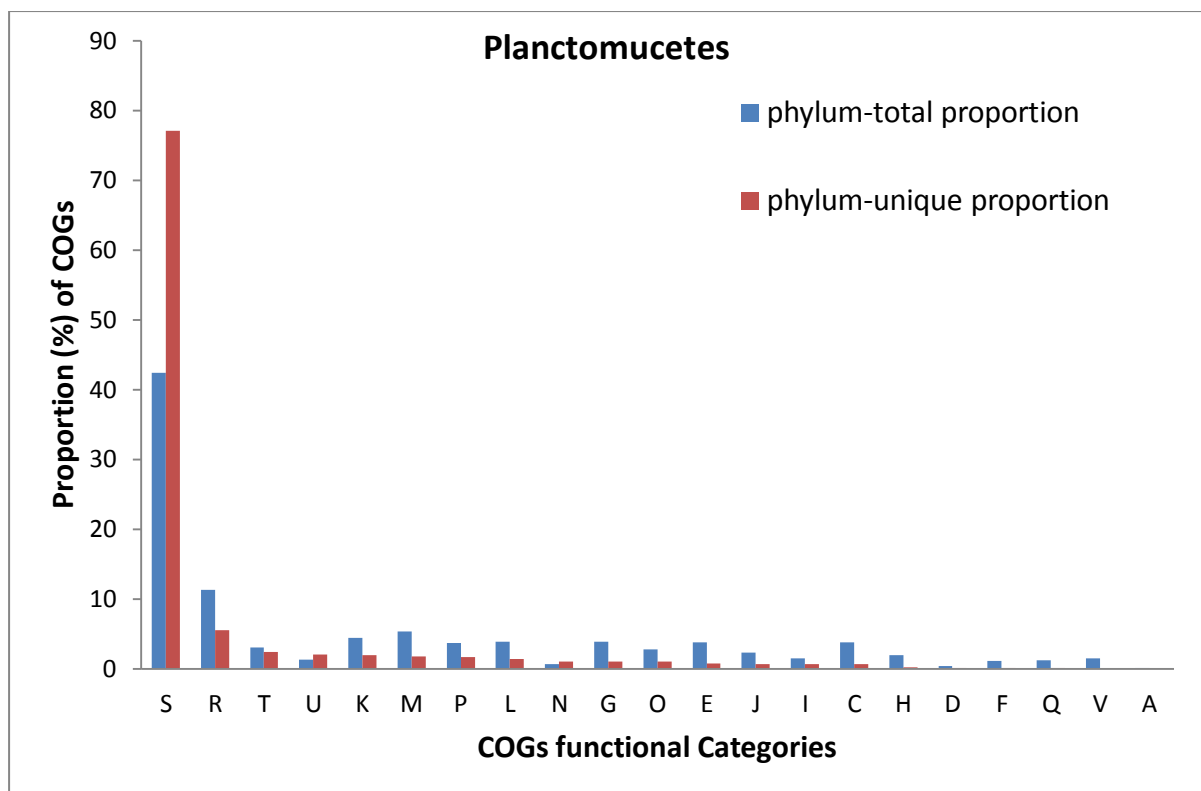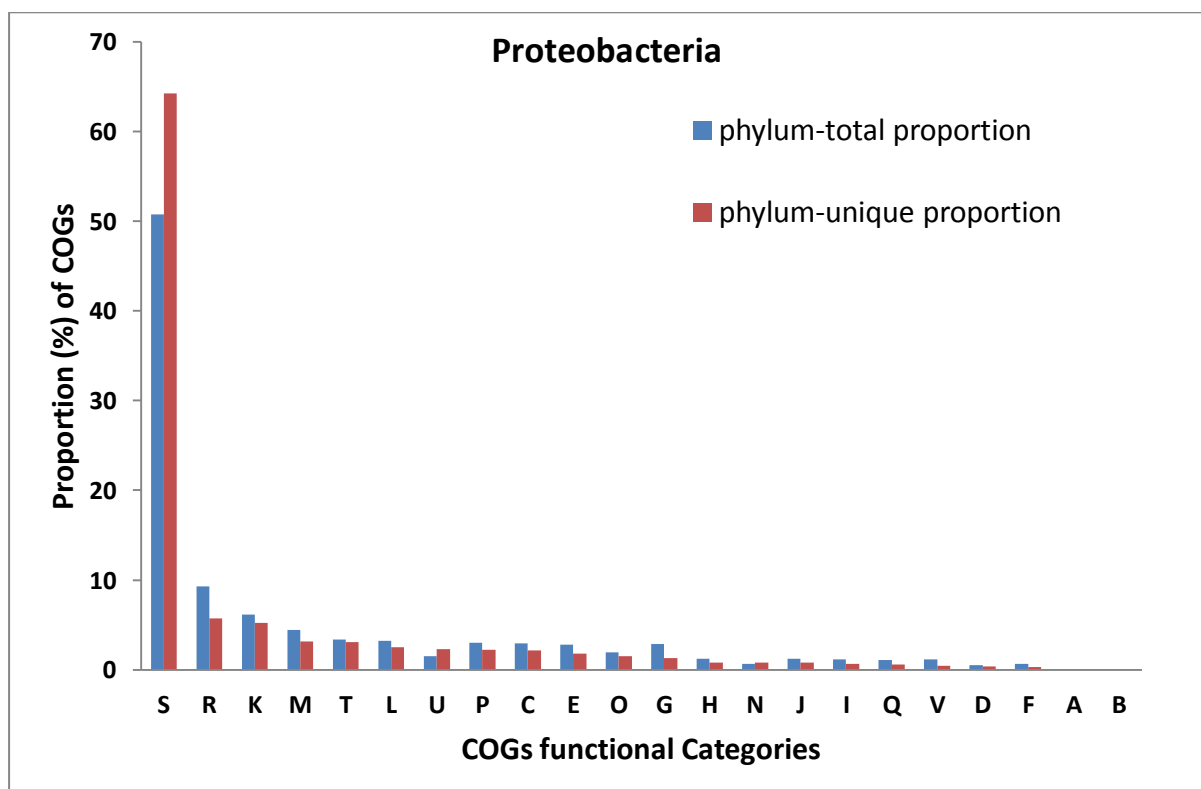

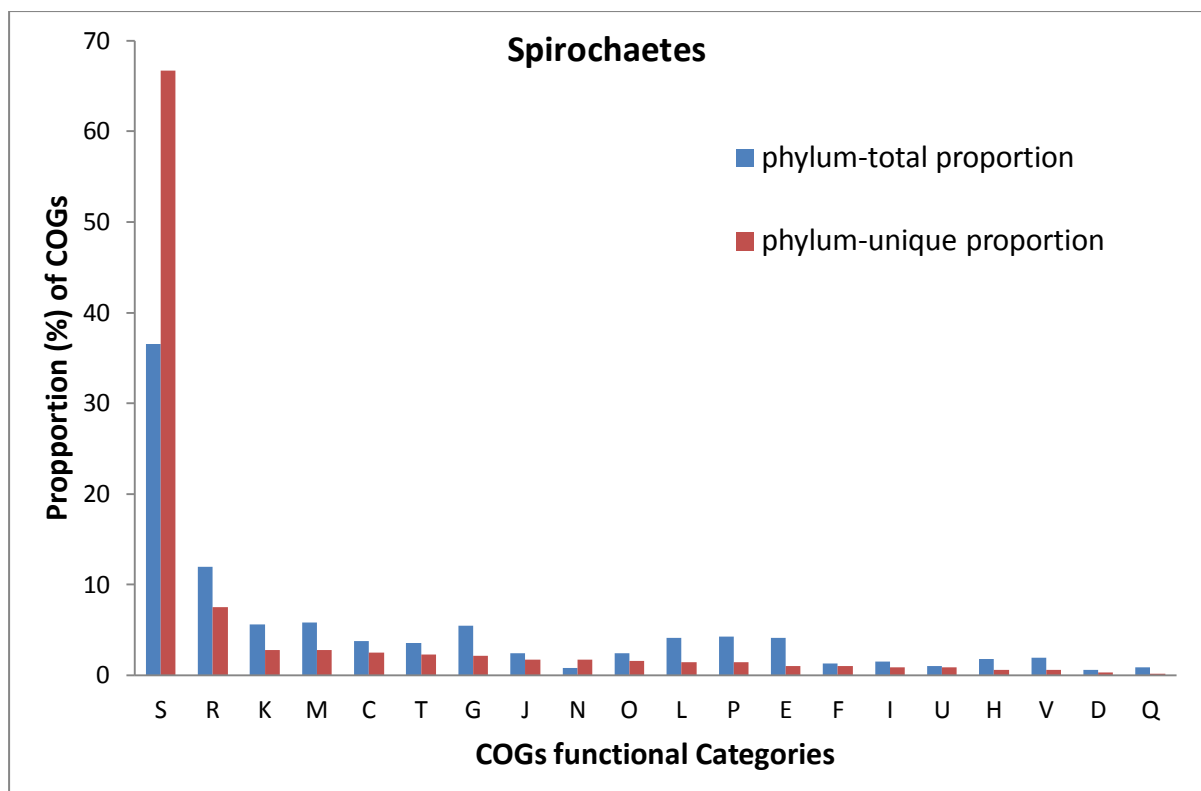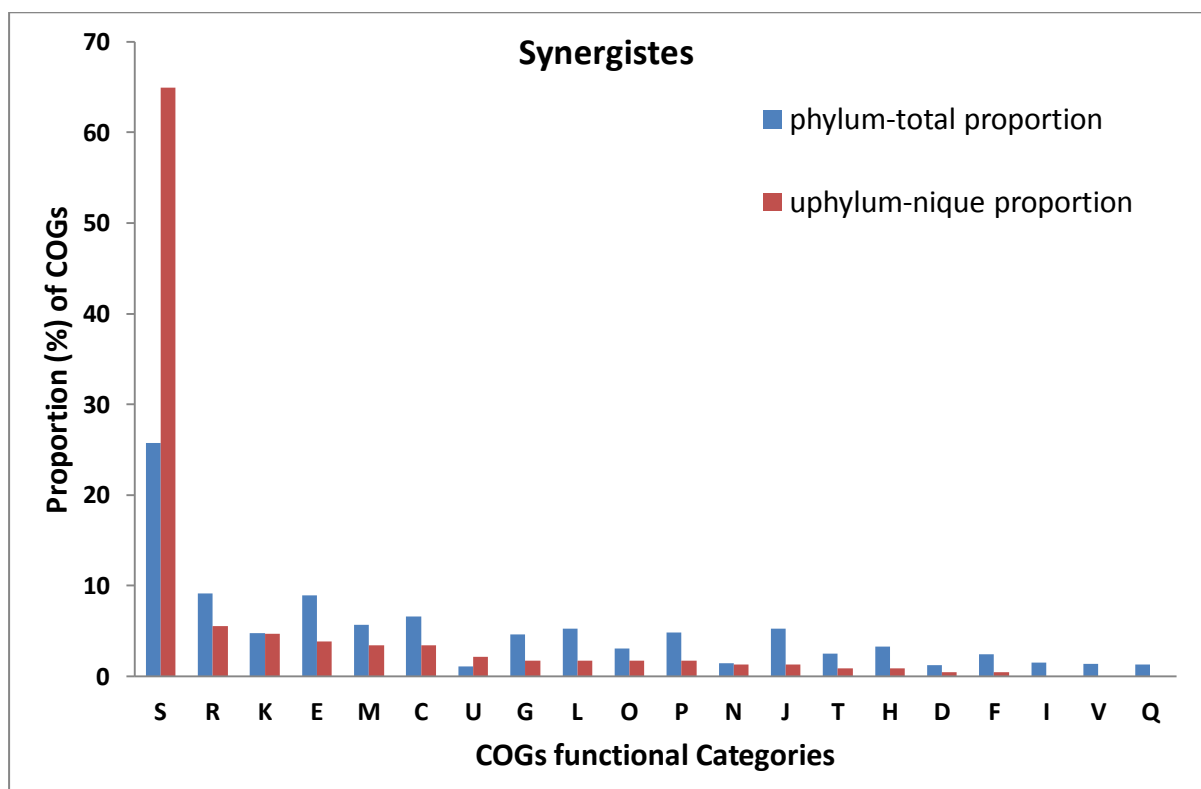

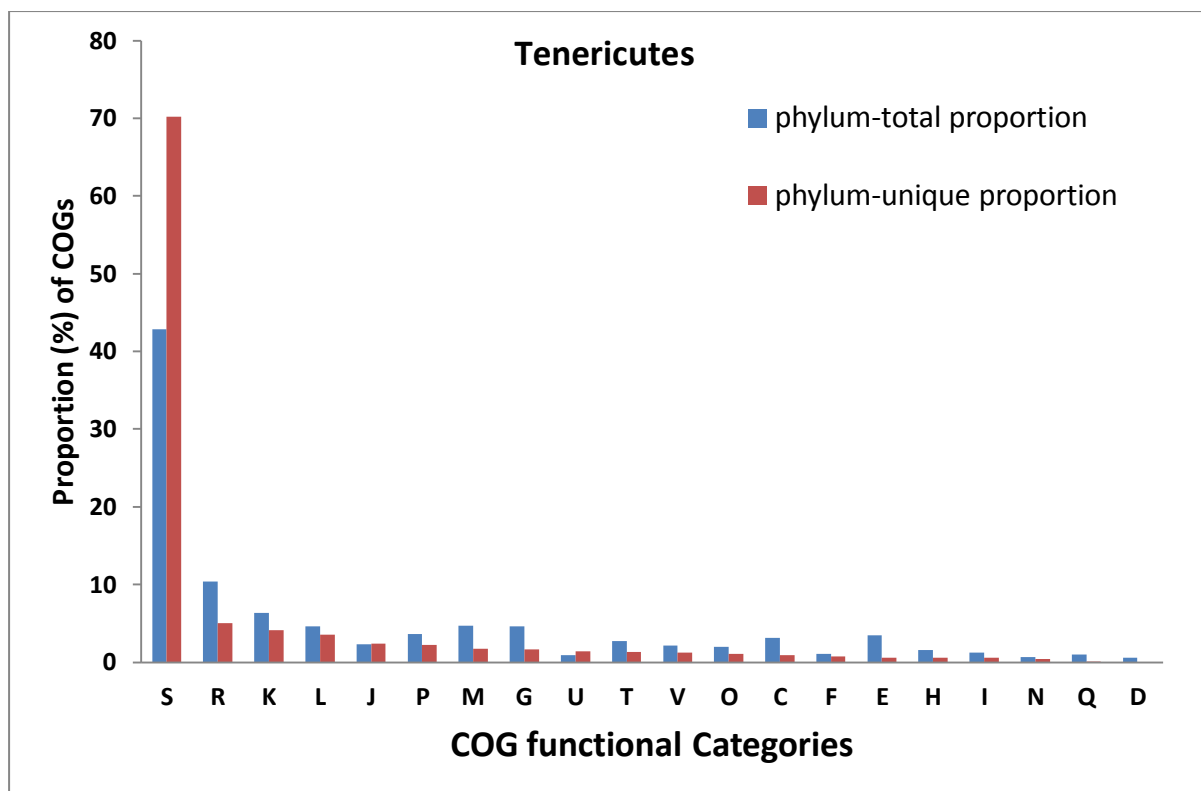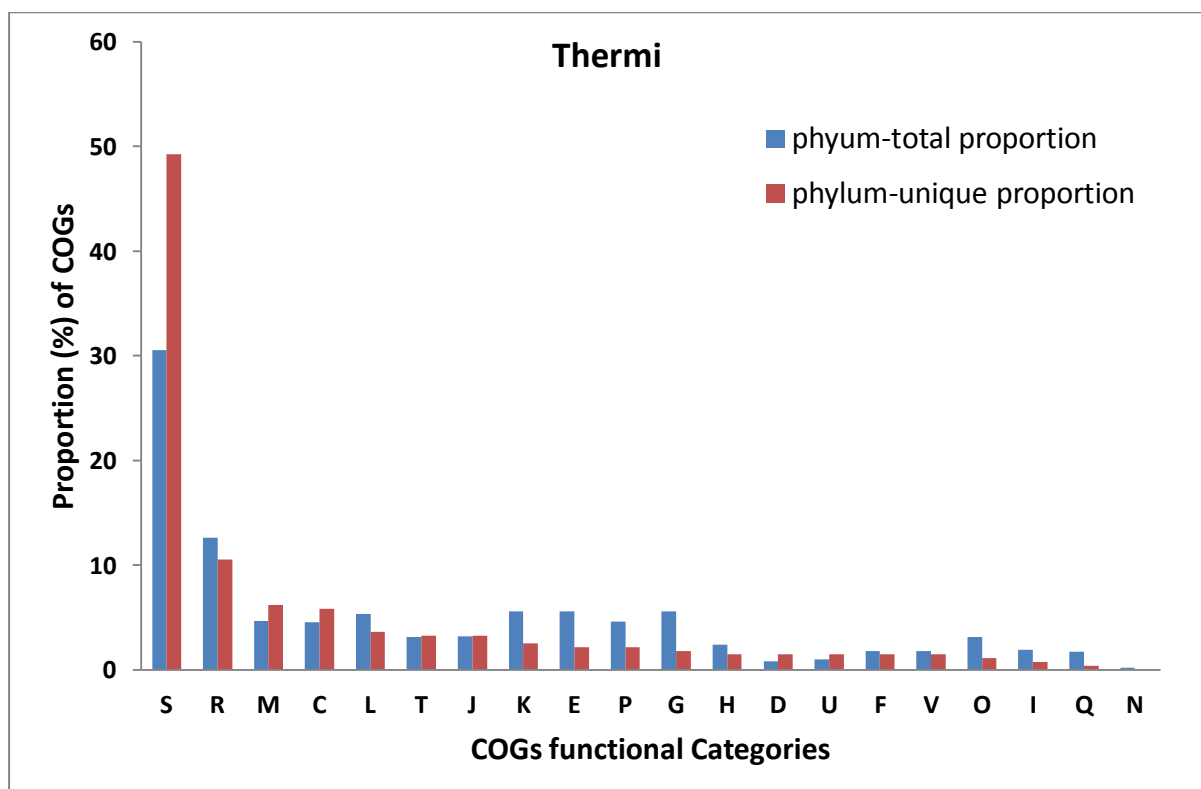

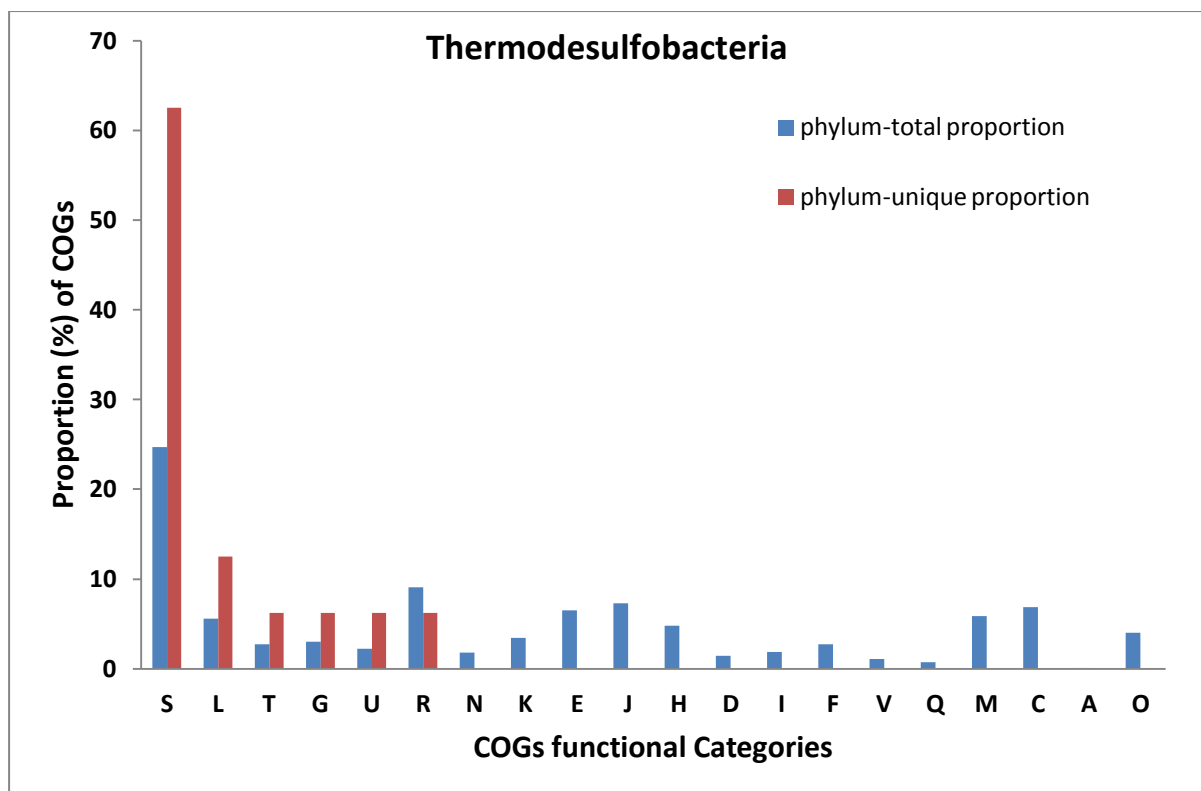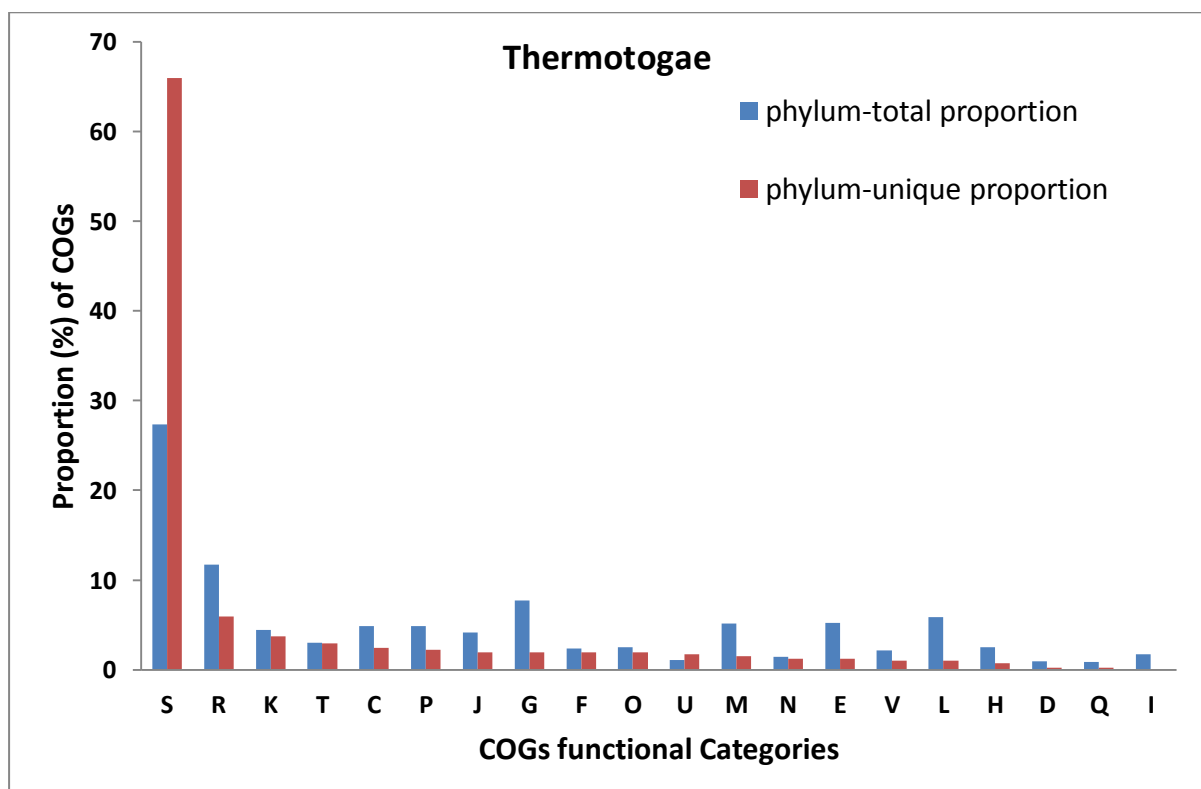

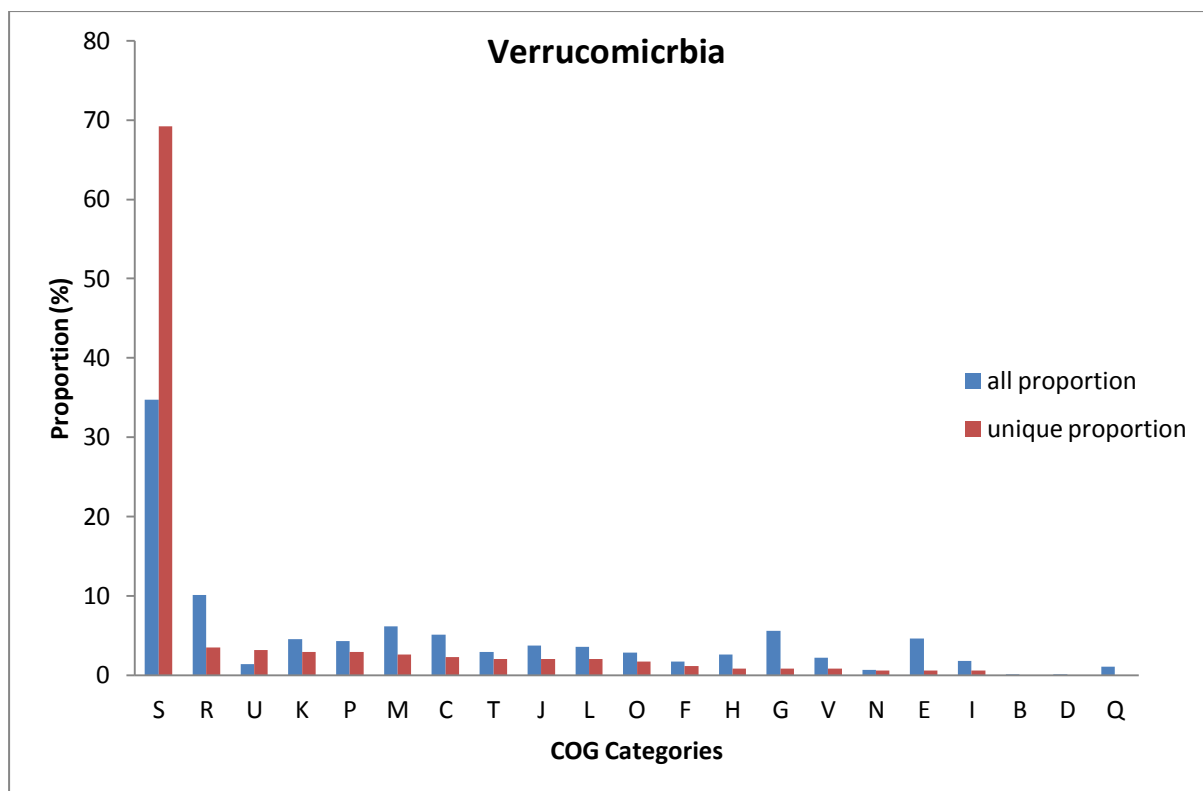

Supplement: Additional file 6: — Proportion of COGs functional categories in phylum-total NOGs and phylum-unique NOGs for all the 27 phyla. [file 12864_2015_1542_MOESM6_ESM.pdf]
